# Socioeconomic disadvantage and polygenic risk of overweight in early and mid-life: a longitudinal population cohort study spanning 12 years

**DOI:** 10.1016/j.lanwpc.2024.101231

**Published:** 2024-11-13

**Authors:** Jessica A. Kerr, Dorothea Dumuid, Marnie Downes, Katherine Lange, Meredith O'Connor, Ty Stanford, Lukar Thornton, Suzanne Mavoa, Kate Lycett, Tim S. Olds, Ben Edwards, Justin O'Sullivan, Markus Juonala, Ha N.D. Le, Richard Saffery, David Burgner, Melissa Wake

**Affiliations:** a Department of Psychological Medicine, University of Otago Christchurch, Christchurch, New Zealand; b Murdoch Children's Research Institute, Parkville, Victoria, Australia; c Department of Paediatrics, University of Melbourne, Parkville, Victoria, Australia; d Alliance for Research in Exercise, Nutrition and Activity, Allied Health and Human Performance, University of South Australia, Adelaide, South Australia, Australia; e Melbourne Graduate School of Education, University of Melbourne, Parkville, Victoria, Australia; f Department of Marketing, University of Antwerp, Antwerp, Belgium; g Environmental Public Health Branch, Environment Protection Authority Victoria, Melbourne, Victoria, Australia; h Melbourne School of Population and Global Health, University of Melbourne, Parkville, Victoria, Australia; i Centre for Social and Early Emotional Development, School of Psychology, Deakin University, Geelong, Victoria, Australia; j Centre for Social Research and Methods, Australian National University, Canberra, Australian Capital Territory, Australia; k The Liggins Institute, University of Auckland, Auckland, New Zealand; l The Maurice Wilkins Centre, University of Auckland, Auckland, New Zealand; m Garvan Institute of Medical Research, Australian Parkinson's Mission, Sydney, New South Wales, Australia; n MRC Lifecourse Epidemiology Unit, University of Southampton, Southampton, United Kingdom; o Singapore Institute for Clinical Sciences, Agency for Science Technology and Research, Singapore; p Department of Medicine, University of Turku, Turku, Finland; q Division of Medicine, Turka University Hospital, Turku, Finland; r Deakin Health Economics, School of Health and Social Development, Deakin University, Geelong, Victoria, Australia; s Department of Paediatrics, Monash University, Clayton, Victoria, Australia

**Keywords:** Socioeconomic disadvantage, Polygenic risk, Adolescent, Adult, Overweight, Obesity, Intervention

## Abstract

**Background:**

We describe BMI by socioeconomic disadvantage and by polygenic risk in parallel cohorts of children and adults (their parents). We examine whether hypothetically intervening to reduce childhood disadvantage could reduce adolescent obesity.

**Methods:**

From a population-based cohort (N = 5107) with a mixed design (survey and direct assessment), 24–31% had genotype data: 1607 children (50% male) followed biennially from age 2–3 to 14–15; 2406 adults (36% male) followed from mean age 35–47 years. Exposures were polygenic risk score for BMI, and neighbourhood- and family-level socioeconomic disadvantage categorised as ‘most’ (top two cohort-specific quintiles), ‘average’, or ‘least’ disadvantage (bottom two quintiles). We explored trends in estimated BMI and risk of overweight/obesity by disadvantage, stratified by polygenic risk. We used generalised linear regression to estimate the reduction in overweight/obesity at 14–15 years in children living in ‘least/average disadvantage’ in early childhood relative to those in ‘most disadvantage’, adjusted for confounders. Causal effect estimates were obtained separately for children with higher and lower polygenic risk.

**Findings:**

A positive trend between disadvantage and overweight/obesity was most apparent among participants with high polygenic risk. Among children with higher polygenic risk (n = 805), hypothetical target trial results imply that intervening to lessen population-wide neighbourhood disadvantage from most to least disadvantage could reduce adolescent overweight/obesity by 32% (risk ratio (RR) 0.68, 95% CI 0.50–0.92), or by 42% if intervening to lessen family disadvantage (RR 0.58, 95% CI 0.42–0.79). Positive effects were smaller when isolating the population to those with lower polygenic risk (7–17%), and for the whole population, regardless of polygenic risk (25–39%).

**Interpretation:**

Children at higher polygenic risk of obesity suffer disproportionate BMI impacts of disadvantage. At the population-level, and especially for those with higher polygenic risk, tackling disadvantage could potentially reduce obesity and associated morbidity, mortality, and costs.

**Funding:**

Australian National Health and Medical Research Council. Funding information is detailed in the funding statement.

**Research in context**

**Evidence before this study**

Obesity is a global problem. Its population-wide health and economic impacts increase as more people live more life-years above an ideal body weight. Adults who have high obesity-specific polygenic risk and who experience socioeconomic disadvantage are at especially high risk of obesity. However, it is not yet known whether these dual forces (polygenic risk and socioeconomic disadvantage) cause higher obesity during childhood itself, which would increase years lived with disability because obesity is usually lifelong. To review the population-based literature examining socioeconomic disadvantage and polygenic predispositions to overweight/obesity among children and adolescents, we conducted a title and abstract search of PubMed for articles published from inception up to August 28, 2023, with no language restrictions, using the following search terms: (adolescen∗ OR youth OR youths OR teen OR teens OR teenage∗ OR child∗ OR juvenile) AND (poverty OR sociodemo∗ OR socioeconomic∗ OR socio-economic∗ OR disadvantage OR deprivation) AND (PRS OR PGS OR genetic OR polygenic OR polygenic score OR polygenic risk score) AND (BMI OR body mass OR body size OR body weight OR overweight OR obes∗) AND (cohort OR follow-up OR followup OR observational OR longitudinal OR prospective). From 103 results we ascertained that four papers, from three population-representative cohort studies (IDEFICS, ECPBHS, TRAILS), have examined the interplay between socioeconomic factors and child/adolescent genetic risk for overweight or obesity. Two papers were most relevant. In the TRAILS cohort, polygenic risk and family socioeconomic status additively predicted overweight development from age 11–26 years. In the IDEFICS cohort, parental education attenuated the impact of children's polygenic risk for developing obesity across two follow-up periods (average period of 6 years) after baseline (mean age 6 years).

**Added value of this study**

Within a population-representative cohort we add value to this literature. We detail the evolution of body mass index (BMI) from earliest childhood (ages 2–3 years) into adolescence (ages 14–15 years) by polygenic risk, socioeconomic disadvantage, and multiple points of biennial measurement. We examine both neighbourhood and family indicators of socioeconomic disadvantage, and describe patterns in a parallel cohort of adults (their parents) across the same period (2006–2018). Further, among genetically vulnerable children, no one has yet estimated the obesity risk potentially averted by reducing population-wide exposure to socioeconomic disadvantage. If living in disadvantage magnifies obesity risk among at-risk groups (e.g., children with genetic vulnerabilities), this provides further reason for direct intervention on population-level disadvantage. Among both children and adults, we observed a trend between increasing levels of socioeconomic disadvantage and increasing risk of developing overweight or obesity over a decade—a trend that was most obvious among participants with high obesity-specific polygenic risk. Moreover, using a target trial framework for causal inference modelling, we show that hypothetical population-wide intervention during early childhood to improve neighbourhood deprivation or to improve family disadvantage (e.g., improving access to education) could reduce the risk of developing adolescent overweight or obesity by 25%–39% (regardless of polygenic risk). This effect was most pronounced in children with high polygenic risk, in whom risk reduction varied between 32% (with neighbourhood-level intervention) and 42% (with family-level intervention).

**Implications of all the available evidence**

In adults, there is previous evidence that polygenic predisposition to obesity can be partially offset by intervention efforts and that those with the highest polygenic risk may benefit the most from population-wide interventions. Hypothetically, we provide the first data to support this in early childhood, a critical period to future outcomes. That is, among children with high polygenic risk, the relationship between socioeconomic disadvantage and presentation of overweight or obesity is greater than it is for those with low polygenic risk. Beyond our hypothetical target trial setting, future research should explore whether directly intervening on early-life disadvantage could reduce the risk of vulnerable adolescents entering adulthood with established obesity, and thence reduce associated inequalities in non-communicable disease. A triple dividend.

Future research should examine system-wide programmes that support governments in implementing policies and interventions to improve disadvantaged neighbourhoods and relieve family economic hardships. Governments can invest in community wealth building programmes, and utilise various policy levers including urban planning requirements, healthy home requirements, subsidised supermarkets, tax policies, and incentives for parents to return to the workforce. Family-level interventions could reduce barriers to accessing health services, healthy and safe housing, education, and employment by addressing hardship and structural obstacles through measures like job creation, educational engagement, and increasing household income (e.g., tax benefits or cash transfers). Focused on genetically vulnerable children, our findings highlight the importance of now examining their exposure to worsening socioeconomic inequalities, cumulative social risks, and the intersections between their exposure to socioeconomic disadvantage and other forms or marginalization (e.g., ethnicity or disability).

## Introduction

Clinical presentations for comorbidities related to overweight and obesity are increasing, and current intervention strategies targeting obesity itself have limited long-term success.^1^ The value of multifaceted population-level obesity prevention is increasingly recognised.^2^ Obesity is influenced by multiple genes involved in metabolism and energy balance, with BMI heritability estimated at 30–40%.^3^ There is also a marked social gradient for many adverse health outcomes, including obesity.^4^^,^^5^ Socioeconomic disadvantage may directly shape emerging phenotypes via epigenetic mechanisms,^6^ and also indirectly result in individuals being at disproportionately higher risk of experiencing poor mental health and obesogenic factors (e.g., inadequate greenspace, healthy foods, resource distribution, health services),^7^ which can magnify polygenic predispositions to obesity.^8^^,^^9^

Increasingly strong evidence supports this paradigm. Among adults, twin and population-representative cohorts show that polygenic factors for obesity have a greater impact among those exposed to low income or low education levels, or among those experiencing neighbourhood deprivation.^8^^,^^10^ Across the life course, data from several birth cohorts show that low, or worsening, socioeconomic status (e.g., education, income) from childhood to adulthood amplifies the polygenic influence of obesity on adulthood phenotypes.^11^ Data from the 1946 British birth cohort (n = 2677)^10^ suggest that polygenic risk and socioeconomic position (primary measure: paternal occupational class) relate independently to higher BMI across the life course (aged 2–69 years); effects were very small within childhood, noting the vastly different social milieu operating 70 years ago when these participants were children. Two contemporary European cohorts indicate higher risk of obesity in young people with higher polygenic risk and poorer family socioeconomic status.^12^^,^^13^ In the TRAILS cohort (n = 1675, studied longitudinally for 15-years following age 11), higher polygenic risk and lower socioeconomic status (composite of parent income, education, occupation) additively predicted an overweight trajectory during adolescence.^12^ In the IDEFICS cohort of children (n = 3098, aged 2–9 years at baseline), higher parental education partially attenuated a polygenic risk for developing obesity, but follow up was short (6 years).^13^ This limited population-based literature relating to children and adolescents is concerning.^10^, ^11^, ^12^, ^13^, ^14^ Consequently, it is unclear at what age the association between polygenic risk and socioeconomic disadvantage emerges and whether effects are stronger for specific sources of socioeconomic disadvantage—both of which are important for translation to intervention.

While there are many reasons to reduce socioeconomic disadvantage, understanding how and when it may amplify the genetic risk of obesity could provide a powerful and less stigmatising economic impetus to do so. Large genotyped and phenotyped population cohorts now enable the use of polygenic risk scores to examine how family and neighbourhood socioeconomic disadvantage shape the magnitude of polygenic associations with one's developing BMI over time and at different life course stages (i.e., childhood and adulthood). This paper draws on two parallel cohorts of children and adults (their parents) over a 12-year period of 7 biennial repeated waves of the population-representative Longitudinal Study of Australian Children (LSAC) to address two aims. Aim 1 was to describe trends in estimated BMI and overweight/obesity risk across childhood and mid-adulthood by socioeconomic disadvantage (at both family and neighbourhood levels), stratified by level of polygenic risk for high BMI. Aim 2 was to estimate the causal effect of hypothetically intervening to lessen socioeconomic disadvantage in early (2–3 years) or in late (12–13 years) childhood on later adolescent BMI and overweight/obesity (14–15 years) among children with higher (vs. lower) polygenic risk. This second aim focused on the child cohort only, because obesity intervention is most impactful if achieved early in the life course.

## Methods

### Participants & procedure

The nationally-representative longitudinal LSAC is Australia's only cohort study that follows the development and life course trajectories of children and their families from all Australian states and territories. Designed to provide an evidence base for service delivery and policy priorities related to the lifelong wellbeing of children born early in the new millennium,^15^ LSAC is conducted by the Australian Institute of Family Studies (AIFS) and the Australian Department of Social Services (DSS). DSS manages LSAC on behalf of the Australian Government, and AIFS manages the design and study content, instrument development, data collection, and sample management. Data collection is conducted by AIFS-trained interviewers.

We utilised data from the Birth (B) cohort of LSAC, whose study design incorporates frequent and ongoing questionnaire-based data collection, linkage to parent and child administrative datasets (not part of this paper), and open data access for researchers.^16^ Because LSAC was designed as a longitudinal study of children's development, the sampling unit of interest was the child. LSAC sampling was led by Growing up in Australia's Sampling Design Team, in partnership with Australia's Health Insurance Commission (Medicare) and the Australian Bureau of Statistics.^16^ Infants were sampled in 2004 using a two-stage clustered random sampling design to be representative of Australia's population. In stage 1, 10% of Australian postcodes were randomly selected (stratified by state, and by urban/rural location); in stage 2, infants aged 0–1 years were selected from Australia's healthcare database (Medicare). Medicare is a universal Australian government programme that covers or reimburses medical costs, 98% of children are registered with Medicare by age 1.^16^ Fifty-seven percent (n = 5107) of invited participants were recruited, and the baseline sample was representative of Australian children (citizens and permanent residents) born between March 2003 and February 2004.^17^ Consenting families were then followed biennially via interviewer home visits to the child and primary parent, at which data were collected on our key exposures. Over the eight LSAC assessment waves, data collection methods included home visits for direct assessments (e.g., measurement of child height and weight), face-to-face interviews with parents and with children themselves (when older) by trained interviewers, and audio computer-assisted interviews.^17^ Trained interviewers repeatedly collected information on a range of socioeconomic factors, lifestyle, and health measures. Specific protocols are described in Table 1 and Supplementary Table S2, with extensive detail in the public space (see Data Sharing Statement). These visits were supplemented by mail-back and online surveys from the second parent.^16^ Of the original 5107 infants, 3764 (74%) were retained until Wave 6 in 2014, and 3127 (61%) were retained to Wave 8 in 2018 at age 14–15 years. This paper draws on data from Waves 2–8, covering 12 years from ages 2–3 to 14–15 for children and from mean ages 35 to 47 for adults (parents).

**Table 1:** Measures table.

Construct	Measure	Collection		Brief protocol
*Exposures*		**LSAC** (Waves 2–8)	**Check Point** (Wave 6.5)	
Disadvantage	Neighbourhood	•		Neighbourhood disadvantage was measured with the census-based Socio-Economic Indexes for Areas (SEIFA) Index of Relative Socioeconomic Disadvantage (IRSD) which is updated every four years based on Australian Census data.^18^ The IRSD is based on the statistical area (SA1) where the child's family live and is a weighted combination of census-collected variables that indicate social and material disadvantage of the neighbourhood (e.g., % of people unemployed, % of occupied private dwellings with no cars). Because the study period spans three census waves (2006, 2011, 2016), the IRSD census variables and the SA1 boundaries are slightly different over time. At each time point, scores are standardised to have a mean of 1000 (national average) and a standard deviation of 100; low scores indicate high disadvantage, with higher scores indicating less disadvantage. In the main analyses, IRSD is converted into cohort-specific quintiles with 1 being high neighbourhood disadvantage, and 5 being low neighbourhood disadvantage (i.e., better/advantaged IRSD). Guided by previous research,^19^ for Aim 2 analysis, we then categorised as ‘most disadvantaged’ (quintile 1–2), ‘average’ (quintile 3), or ‘least disadvantaged’ socioeconomic conditions (quintile 4–5).
	Family	•		Family disadvantage (socioeconomic position (SEP))^20^ was a composite of parent/adult-reported combined household income, current or most recent occupation of each parent, and highest achieved educational qualification of each parent. Each component was scaled, and an unweighted average calculated over 3 values in a single-parent household or over 5 values in a dual-parent household. The unweighted average variable at each LSAC wave was standardised within the wave (mean 0, SD 1); low z-scores indicate high disadvantage, with higher z-scores indicate less disadvantage. In the main analyses, SEP is converted into cohort-specific quintiles with 1 being high family disadvantage, and 5 being low family disadvantage (i.e., better/advantaged SEP). For Aim 2 analysis, we then categorised as ‘most disadvantaged’ (quintile 1–2), ‘average’ (quintile 3), or ‘least disadvantaged’ socioeconomic conditions (quintile 4–5).
Genetic Risk	Polygenic risk score (PRS) for BMI		•	Participants' genetic samples were isolated using Illumina Infinium® Global Screening Array-24 v1.0. Following which the PRS was developed with a scoring algorithm derived from a genome-wide association study (GWAS) of 340,000 participants using Khera et al.‘s methods.^21^ To create the PRS,^22^ we matched 2,048,277 individual nucleotide polymorphisms from CheckPoint participants to the GWAS, and then multiplied the number of risk alleles for these variants by estimated effect sizes. These values were summed to create a PRS for each participant that is a proxy for polygenic risk for high BMI in the past, present, and future. Following the creation of the PRS, we derived genetic principal components from a Principal Components Analysis of the CheckPoint genetics dataset. Because the PRS was created with summary data from European populations,^21^ to derive variables to control for population structure (i.e., systematic differences between different sub-populations within the cohort, see Supplementary Table S1 for further detail) for our causal analysis (Aim 2), we selected the top five components based on a scree plot and initial regression models showing that further components did not contribute to the models.^22^ This PRS accounts for 12% of the variance in BMI z-scores within CheckPoint children (11–12 years), and 9% of the variance in CheckPoint adults BMI.^22^ For Aim 1 analyses, PRS is converted to quintiles with 1 being lower risk, and 5 being higher risk; for Aim 2 analysis the PRS is divided at the median to create two groups indicating lower vs. higher polygenic risk.
*Outcomes*		**LSAC** (Waves 2–8)	**Check Point** (Wave 6.5)	
Body Mass Index Overweight and Obesity Status	Objective height & weight (child) Self-reported height & weight (adult/parent)	•		During LSAC home visits children's height and weight were measured by trained interviewers. Weight was measured in light clothing without shoes using HoMedics digital scales (Waves 2–3), or Tanita body fat scales (Waves 4–8). Height was measured to the nearest 0.1 cm using an Invicta stadiometer (Waves 2–3), or a laser stadiometer (Waves 4–8). Two measurements were taken, and a third if these differed by > 0.5 cm; the average of the two closest measures was used. Adults' height and weight were self-reported at all waves. For the main analysis raw BMI was calculated as weight(kg)/(height(m)^2^) and for children we also provide z-scores for sample characteristics (Table 2) according to the US Centers for Disease Control (CDC) reference values. For main analysis, CDC cut-offs were used to determine overweight/obesity among children/adolescents at the ≥85th percentile; among adults overweight/obesity was BMI ≥25 kg/m^2^.

Abbreviations: LSAC: Longitudinal Study of Australian Children; SEP: socio-economic position; SEIFA: Socio-Economic Index for Areas; IRSD: Index of Relative Socioeconomic Disadvantage; PRS: polygenic risk score; GWAS: Genome-Wide Association Study; CDC: Center for Disease Control.

**Fig. 1:**
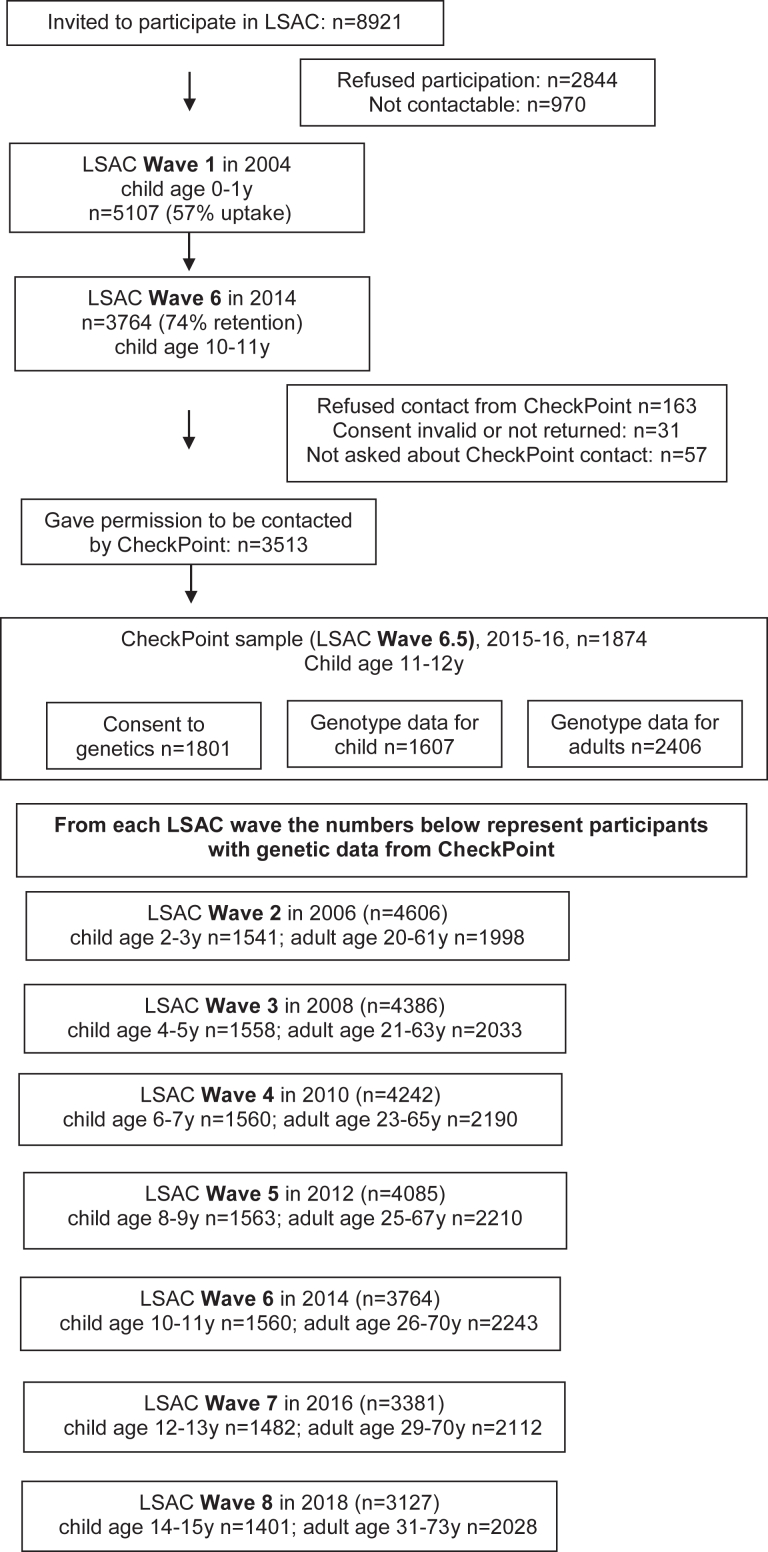
**Participant flow chart through LSAC to CheckPoint**.

A nested biophysical module, the Child Health CheckPoint, enabled the collection of genetic data. Fig. 1 illustrates that 3513 of the LSAC families who participated in Wave 6 gave consent to be contacted by our CheckPoint team and to be invited to participate in this nested module.^23^ Families were re-recruited for this optional module by mail and telephone in 2015 between LSAC Waves 6 and 7. This module was offered to all children and one of their parents at child age 11–12 years^23^; 1874 children (37% of baseline cohort, 50% of Wave 6 participants) took part from February 2015 to March 2016 (Fig. 1). Most CheckPoint families lived in major cities, and the distribution of families across Australia's states and territories is similar to the Australian population and similar to the initial LSAC sample.^23^ CheckPoint offered a visit to its full Assessment Centre in Australia's 7 largest cities, a condensed Centre visit in 8 regional towns, or a shorter home visit. A general 1.5–3.5 h health assessment comprised 15-min “stations” (protocol described elsewhere^23^). For DNA extraction from the study child and attending parent, venous blood, dried blood spots, and saliva samples were collected at Assessment Centres, with dried blood spots and mouth swabs (Oracollect DNA OCR-100) collected at home visits. For children with more than one parent, a second parent was able to return a mouth swab by post. The protocol, DNA collection, and extraction methods are detailed in Lange et al.^22^ Only participants with genotype data (N = 1607) were included in this study.

CheckPoint protocols were approved by The Royal Children's Hospital (Melbourne, Australia) Human Research Ethics Committee (33225D) and the Australian Institute of Family Studies Ethics Committee (14–26), which also approved LSAC. Prior to participating in the CheckPoint assessments, the attending parent/guardian provided written and verbal informed consent (to a trained research assistant) for their own and their child's participation. All data and samples were stored in a deidentified manner.^23^

### Measures

In Table 1 we describe both measures of family and neighbourhood socioeconomic disadvantage, detail the BMI polygenic risk score (PRS), and the two outcome variables BMI and overweight/obesity. Data on BMI and indicators of disadvantage were obtained during Waves 2–8 of LSAC during 2006–2018. The collection of specimens for DNA (venous blood, dried blood spot, buccal swap, or saliva) for the creation of the PRS was part of CheckPoint data collection in 2015–2016.^22^

### Statistical analyses

Children and adults were part of the descriptive Aim 1, but only children were part of the main causal analysis in Aim 2. Participants in each of the child and adult cohorts were included in the corresponding analysis sample if they had a non-missing PRS for BMI and at least one non-missing BMI measurement across LSAC Waves 2–8. On average, 95% of this child cohort had non-missing BMI, compared to 88% of the adult cohort. Because missing data were minimal for the main analysis (≤5% for children in Aim 2), and because the complete CheckPoint cohort and our analytic samples (e.g., Supplementary Table S3) did not meaningfully differ on key demographics (e.g., disadvantage, BMI), we did not pursue multiple imputation, but survey weights were applied to the main analysis to account for potential sectional bias in the analysis sample (see Aim 2 below).

For Aim 1, R (v4.2.2) and the glmmTMB package (v1.1.5) were used to fit all mixed effect models. Trends in BMI and overweight/obesity status across childhood were examined by fitting generalised linear mixed models adjusted for sex (identity link function for continuous BMI and log-link function for binary overweight/obesity indicator). Because Aim 1 was descriptive, no other covariate adjustments or adjustments for multiple testing were made.^24^, ^25^, ^26^ Fixed effects included age (categorised into 2-year age bands per LSAC Wave), and, as guided by previous research,^19^ cohort-specific socioeconomic disadvantage quintile (neighbourhood or family, Table 1), and the interaction between them. Then, to explore whether trends differed by level of polygenic risk, a polygenic risk variable (quintiles) as well as the two-way interactions with both age and socioeconomic disadvantage quintile (neighbourhood or family) were also added as fixed effects to each model. Random effects included random intercepts to account for repeated measures over time within the participant, and random slopes for LSAC wave. The models were fit using restricted maximum likelihood (REML) and the nlmimb optimiser, assuming an unstructured variance-covariance matrix. Fixed effects were evaluated using Type III Wald χ2 tests.

The same approach was applied to adult models. However, because adult participants had a wide age range at study commencement (mean 35.3 years; range 20–61 years) we categorised adult age as follows: <30 years, 30–35 years, 35–40 years, 45–50 years, >50 years. Furthermore, an additional random intercept for family accounted for there being up to two parents per child (i.e., potential correlation between members of the same family unit), and the random intercept to account for repeated measures over time was within family, rather than participant. The adult models also allowed fitting a wave covariate as age categories contained variance in a wave.

**Fig. 2:**
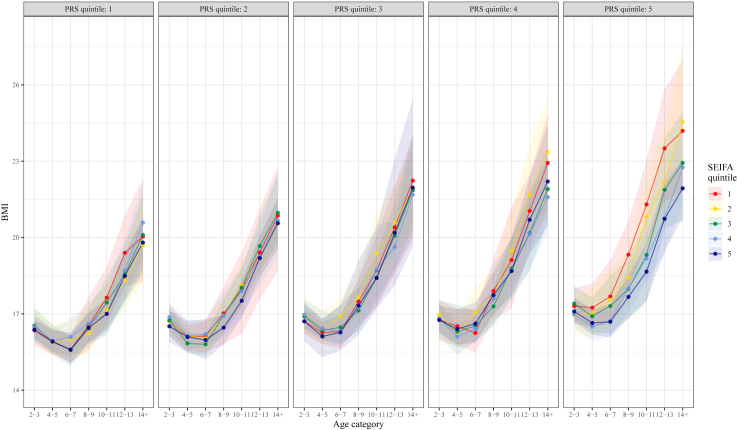
**BMI across childhood by neighbourhood disadvantage (SEIFA) quintile, stratified by PRS quintile**.

**Fig. 3:**
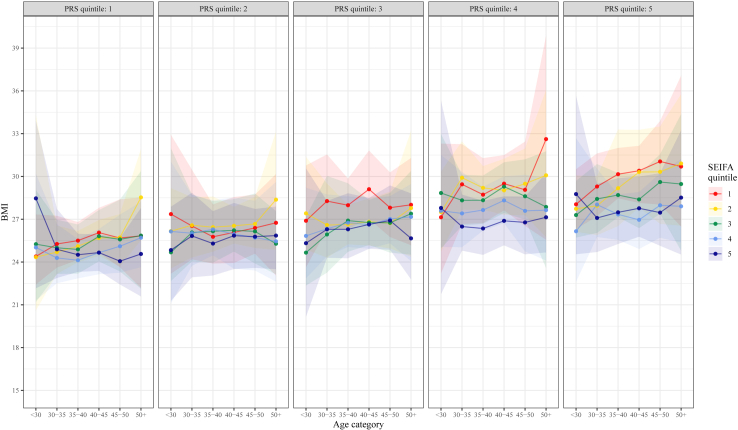
**BMI across adulthood by neighbourhood disadvantage (SEIFA) quintile, stratified by PRS quintile**.

Fitted values (representing mean BMI or risk of overweight/obesity) at each combination of age and disadvantage quintile were plotted (e.g., Supplementary Figure S1), and additionally by each level of polygenic risk (e.g., Figs. 2 and 3) with 95% confidence intervals obtained from R = 1000 bootstrap samples^27^ including conditional random effects, sampled at the participant level with all participant's observations included. Marginal estimates for age and disadvantage quintile strata were derived as the mean estimates over PRS quintile. The supplementary material includes plots of strata predictions and confidence intervals, and output for interaction effects (PRS x disadvantage). Because Aim 1 focuses on describing BMI, or overweight/obesity, trends over time within PRS and disadvantage strata and was not focused on estimating outcomes at particular ages or waves, we did not employ probability sample weighting (i.e., survey weighting) to correct for different response patterns, and the results from Aim 1 should not be interpreted as providing population prevalence of overweight or obesity. R code, model selection considerations, and further model fitting details are available at: https://github.com/tystan/sociopolygenic.

To address the second aim, we used the target trial framework to define in detail the causal effect of interest.^28^, ^29^, ^30^, ^31^, ^32^ This framework involves two important steps. The first is to specify the target trial, defined as the hypothetical randomised experiment that would ideally be implemented to answer our research question. In this initial step we developed a detailed description of the key protocol components of eligibility criteria, treatment strategies, assignment procedures, follow-up period, and outcome (Supplementary Table S1). The second step is then to consider the assumptions under which one may emulate the target trial with the observational data available to obtain an unbiased estimate of the target causal effect. The framework assists with the systematic identification of potential sources of bias to inform statistical analysis planning and appropriate interpretation of findings.

Using Stata/SE version 17, the causal effect of intervening to improve disadvantage in early (2–3 years) childhood on later adolescent overweight/obesity (14–15 years) was estimated using generalised linear regression with a Poisson log-link function with adjustment for identified potential confounders (identified with a directed acyclic graph, Supplementary Figure S11). The adjustment set included age and sex, and the following variables that were available in our dataset and considered to be causes of the exposure or the outcome or both, and not on the causal pathway: Wave 1 (0–1 years) disadvantage, household conflict, parental mental health; and specific to accounting for any genetic biases, the top five genetic principal components and DNA sample type (Supplementary Table S2). Confounding variables that were numerical were included in the model as linear terms. Analyses were stratified by level (i.e., higher vs lower) of polygenic risk based on the median split of the PRS variable. Adjusted risk ratios with 95% confidence intervals were obtained for the least and average levels of socioeconomic disadvantage relative to the most disadvantage. The causal effect of exposure to socioeconomic disadvantage in early childhood on the continuous BMI outcome was also estimated using standard regression with causal effects representing mean differences. Under a number of assumptions, including that the selected set of confounders is a sufficient set to adjust for confounding bias, this adjusted mean difference or adjusted risk ratio can be interpreted as the reduction in BMI and/or risk of adolescent overweight/obesity that could hypothetically be achieved among those experiencing socioeconomic disadvantage if they experienced less disadvantage. These statistics are presented for sub-groups of genetic risk level, with population-wide statistics presented in Supplementary Table S4.

Survey weights were applied to all causal analyses (i.e., Aim 2) using Stata survey procedures. The survey weights were calculated considering the selection probability of each participant and were adjusted for non-response, loss to follow-up and benchmarked to population numbers in major (poststratification) categories of the population of children born in 2004 (i.e., the original recruitment of the baseline Birth cohort in LSAC). More detail on the calculation of weights is provided elsewhere.^33^ Analyses were conducted separately for neighbourhood and family socioeconomic disadvantage, and all analyses were repeated considering exposure to socioeconomic disadvantage (neighbourhood and family) in late childhood (12–13 years; pre-exposure confounders from 10 to 11 years) to explore the impact of intervening at a different time point. Sensitivity analyses were conducted restricting the sample to those of European descent (n = 1365) because the PRS were generated with summary data from European populations.^22^

In accordance with the recommendations of international scientific committees,^34^^,^^35^ we have evaluated effect estimates in terms of their overall direction, magnitude, and precision rather than dichotomous interpretations of statistical significance using a cut point of 0.05.

### Role of the funding source

The funding bodies did not play any role in the study design, collection, analysis, interpretation, manuscript writing, or submission.

## Results

### Sample characteristics

Sample characteristics are shown in Table 2. Most children and adults were of European ancestry at every wave (84–89%). Sex was evenly distributed at all waves for children, whereas adult participants were majority female. Most adults had completed tertiary education and/or completed high school. Over time the proportion of adults who were employed and had an average income over $52,000/year increased steadily. When standardised, the composite of family combined (if dual-parent household, see Table 1) household income/s, occupation/s, highest educational qualification/s resulted in higher-than-average family socioeconomic position (SEP) values. SEP exceeded 0.0 at every wave (range 0.13 to 0.38), indicating slightly higher socioeconomic positioning than the population-derived LSAC cohort at baseline. Similarly, mean neighbourhood disadvantage scores (>1000 SEIFA IRSD, range 1021–1030) and narrower standard deviation (<100) indicates on average a slightly less disadvantaged and more homogeneous sample than the general Australian population.

**Table 2:** Descriptive statistics for demographics, socioeconomic disadvantage and body mass in the analysis sample over the seven waves of data collection, mean (SD) or %.

	Number	Demographics			Indicators of socioeconomic disadvantage					Body mass		
		Age	Male %	% European ancestry^a^	Area SEIFA score	Family SEP z-score	% Average yearly income ≥$52,000	Employed %	Higher education^b^ %	BMI z-score	BMI	Overweight obese %
**Children**												
Wave 2	1541	2.3 (0.4)	50.2	85.1	1021 (58)	0.27 (0.95)	–	–	–	0.5 (1.1)	16.8 (1.5)	31.2
Wave 3	1558	4.2 (0.4)	50.1	84.9	1025 (58)	0.25 (0.94)	–	–	–	0.5 (1.0)	16.3 (1.6)	30.1
Wave 4	1560	6.3 (0.5)	50.4	85.2	1025 (57)	0.23 (0.95)	–	–	–	0.4 (0.9)	16.4 (2.0)	20.0
Wave 5	1563	8.4 (0.5)	50.6	85.0	1026 (60)	0.20 (0.99)	–	–	–	0.3 (0.9)	17.3 (2.5)	20.1
Wave 6	1560	10.4 (0.5)	50.7	84.7	1027 (60)	0.17 (0.98)	–	–	–	0.3 (1.0)	18.6 (3.1)	22.0
Wave 7	1482	12.5 (0.5)	48.3	85.0	1027 (63)	0.15 (0.98)	–	–	–	0.3 (1.0)	20.2 (3.6)	23.5
Wave 8	1401	14.3 (0.5)	50.7	84.5	1027 (63)	0.13 (0.96)	–	–	–	0.3 (1.0)	21.7 (3.9)	24.5
**Adults**												
Wave 2	1998	35.3 (5.0)	37.1	88.4	1024 (57)	0.38 (0.92)	33.6	76.1	77.8	–	25.7 (4.6)	49.5
Wave 3	2033	37.1 (5.1)	34.0	88.6	1026 (58)	0.35 (0.93)	35.8	78.9	77.9	–	26.1 (4.9)	52.3
Wave 4	2190	39.2 (5.1)	33.2	87.9	1026 (57)	0.32 (0.94)	41.0	81.4	78.3	–	26.5 (5.4)	55.1
Wave 5	2210	41.1 (5.1)	31.9	88.0	1028 (59)	0.27 (0.97)	46.4	84.6	77.9	–	26.8 (5.6)	56.6
Wave 6	2243	43.1 (5.1)	34.3	87.8	1029 (60)	0.25 (0.96)	52.1	86.2	78.3	–	27.1 (5.6)	58.6
Wave 7	2112	45.1 (5.0)	31.1	88.4	1030 (62)	0.23 (0.96)	57.2	88.0	79.0	–	27.6 (6.1)	61.3
Wave 8	2028	47.1 (5.1)	31.4	88.1	1030 (62)	0.20 (0.95)	64.5	90.0	79.8	–	27.8 (6.0)	64.4

Child mean PRS (n = 1607): 18.9 (SD 0.08). Adult mean PRS (n = 2406): 18.9 (SD 0.08).

Family SEP z-scores below 1 are more disadvantaged, and z-scores above 1 are considered less disadvantaged.

Area neighbourhood SEIFA IRSD scores below 1000 are considered more disadvantaged (i.e., a high proportion of relatively disadvantaged people in this area), and areas with scores above 1000 are considered less disadvantaged.

Across the seven waves, average missingness was 5% for child BMI, 2.5% for child disadvantage, 12% for adult BMI, 3% for adult disadvantage.

Abbreviations: SD, Standard Deviation; SEP, Socio-economic Position; SEIFA, Socio-Economic Indexes for Areas; IRSD, Index of Relative Socioeconomic Disadvantage; BMI, body mass index.

a Genetic ancestry was determined by a Principal Components Analysis of our cohort genetic data merged with a reference sample with ancestries defined. Further detail is available in Lange et al.^22^

b Higher education is the % who obtained bachelor's degree or higher and/or completed high school to Year 12.

Children's average BMI increased with age, reaching a mean of 21.7 kg/m^2^ by age 14–15, yet the percentage with overweight/obesity decreased from 31.2% at age 2–3 years to 24.5% at age 14–15 years. Among adults, the average BMI increased steadily over the 12-year follow-up, from 25.7 kg/m^2^ to 27.8 kg/m^2^; the percentage with overweight/obesity (BMI >25 kg/m^2^) increased from 49.5% in Wave 2 (mean age 35.3 years) to 64.4% by Wave 8 (mean age 47.1 years).

**Aim 1: Trends in estimated BMI by socioeconomic disadvantage, stratified by polygenic risk** (see Supplement for additional figures and estimates).

From ages 6–7 years into later childhood and adolescence, we appeared to observe a trend between greater socioeconomic disadvantage and higher BMI (Supplementary Figure S1). Despite nonsignificant interaction effects (Supplementary Tables S7–S14), this trend was most apparent among children with high polygenic risk (e.g., Fig. 2). Compared to neighbourhood disadvantage (Fig. 2), the trend between higher disadvantage and greater BMI appeared to emerge at lower levels of polygenic risk for family-level socioeconomic disadvantage (Supplementary Figure S2). Similar to patterns described in the child cohort, among adults we appeared to observe that the same trend between greater socioeconomic disadvantage and higher estimated BMI was consistent throughout adulthood (Supplementary Figure S6), and most obvious at the highest levels of polygenic risk (e.g., Fig. 3).

**Aim 2: Causal effects of intervening to improve socioeconomic disadvantage during childhood on adolescent BMI outcomes**.

Unstratified population-wide results are available in Supplementary Table S4, and show that regardless of polygenic risk, population-wide intervention to improve disadvantage could reduce the risk of adolescent overweight or obesity by 25%–39%. Results stratified by level of polygenic risk are presented in Table 3. Exposure to family or neighbourhood socioeconomic disadvantage had a sizeable influence on adolescent BMI outcomes in children with higher polygenic risk but effects were smaller in magnitude among children with lower polygenic risk.

**Table 3:** Estimated causal effect of childhood disadvantage on adolescent BMI and overweight/obesity adjusted for potential confounders.

Outcome:	Overweight or Obesity at 14–15 years				Body Mass Index at 14–15 years			
Exposure to neighbourhood disadvantage (SEIFA, IRSD) at:	Early Childhood ages 2–3		Late Childhood ages 12–13		Early Childhood ages 2–3		Late Childhood ages 12–13	
	RR (95% CI)	p	RR (95% CI)	p	MD (95% CI)	p	MD (95% CI)	p
**High Polygenic Risk Score (PRS)**								
**Compared to living in disadvantage**								
Living in average disadvantage	1.02 (0.75, 1.38)	0.90	0.89 (0.61, 1.30)	0.54	−0.00 (−0.05, 0.05)	0.89	−0.03 (−0.08, 0.02)	0.28
Living in least disadvantage	0.68 (0.50, 0.92)	0.01	0.78 (0.54, 1.12)	0.17	−0.02 (−0.06, 0.02)	0.39	−0.05 (−0.09, −0.01)	0.03
**Low Polygenic Risk Score (PRS)**								
**Compared to living in disadvantage**								
Living in average disadvantage	1.09 (0.64, 1.86)	0.75	1.21 (0.71, 2.05)	0.49	0.01 (−0.03, 0.05)	0.65	0.01 (−0.04, 0.06)	0.63
Living in least disadvantage	0.93 (0.55, 1.59)	0.79	1.34 (0.74, 2.41)	0.33	−0.01 (−0.04, 0.03)	0.67	0.01 (−0.03, 0.05)	0.63

**Exposure to family disadvantage (SEP) at:**	Early Childhood ages 2–3		Late Childhood ages 12–13		Early Childhood ages 2–3		Late Childhood ages 12–13	
	**RR (95% CI)**	**p**	**RR (95% CI)**	**p**	**MD (95% CI)**	**p**	**MD (95% CI)**	**p**

**High Polygenic Risk Score (PRS)**								
**Compared to living in disadvantage**								
Living in average disadvantage	0.88 (0.61, 1.26)	0.48	0.70 (0.46, 1.08)	0.11	−0.04 (−0.09, 0.01)	0.15	−0.03 (−0.08, 0.01)	0.16
Living in least disadvantage	0.58 (0.42, 0.79)	0.001	0.63 (0.45, 0.87)	0.01	−0.06 (−0.10, −0.02)	0.01	−0.04 (−0.09, −0.00)	0.03
**Low Polygenic Risk Score (PRS)**								
**Compared to living in disadvantage**								
Living in average disadvantage	0.92 (0.51, 1.67)	0.79	1.15 (0.60, 2.19)	0.68	−0.02 (−0.06, 0.02)	0.39	−0.01 (−0.05, 0.04)	0.76
Living in least disadvantage	0.83 (0.49, 1.43)	0.50	0.87 (0.54, 1.41)	0.58	−0.03 (−0.06, 0.01)	0.17	−0.03 (−0.07, 0.01)	0.13

SEIFA-specific intervention models adjusted for sex, age, genetic principal components, genetic collection/sample type, and confounders measured 2 years prior to the exposure: *family-level disadvantage* (SEP: parent income, occupation, education), family household conflict, parental mental health.

SEP-specific intervention models adjusted for sex, age, genetic principal components, genetic collection/sample type, and confounders measured 2 years prior to the exposure: *neighbourhood-level disadvantage* (SEIFA, IRSD), family household conflict, parental mental health.

High polygenic risk defined at median split. Survey weighting applied to all analysis. RR: risk ratio; MD: mean difference; SEIFA: Socio-Economic Indexes for Areas; IRSD: Index of Relative Socioeconomic Disadvantage; SEP: socioeconomic position; CI: confidence interval.

Disadvantage: Quintile 1–2; Average Disadvantage: Quintile 3; Least Disadvantage: Quintile 4–5.

#### Neighbourhood disadvantage

Of those with higher polygenic risk (top 50%), 41–44% of children living in most and average neighbourhood disadvantage at ages 2–3 years had overweight/obesity by 14–15 years, compared with 27% of those living in the least disadvantaged neighbourhood (Supplementary Table S5). For children with lower polygenic risk (bottom 50%), 14–18% had overweight/obesity by 14–15 years across all levels of neighbourhood disadvantage.

For children with higher polygenic risk (Table 3), hypothetical intervention during early childhood (at 2–3 years) to improve neighbourhood socioeconomic conditions from most to least disadvantage was estimated to reduce the risk of adolescent obesity by 32% (adjusted RR 0.68, 95% CI 0.50–0.92), even though such hypothetical intervention was estimated to only shift BMI by a nonsignificant −0.02 kg/m^2^ (95% CI −0.06 to 0.02). Effects on the outcomes were similar if neighbourhood disadvantage was hypothetically reduced during later childhood at 12–13 years (Table 3).

#### Family disadvantage

Table 3 shows the estimated effects of family disadvantage on overweight/obesity status and BMI for children with higher and lower polygenic risk. Among those with higher polygenic risk, hypothetical intervention during early childhood (at 2–3 years) to improve family socioeconomic circumstances from most to least disadvantage was estimated to reduce the risk of adolescent overweight/obesity by 42% (adjusted RR 0.58, 95% CI 0.42–0.79), and shift BMI by −0.06 kg/m^2^ (95% CI −0.10 to −0.02). In comparison, this same hypothetical intervention (i.e., improving family socioeconomic circumstances from most to least disadvantaged levels) among children with lower polygenic risk was estimated to reduce the risk of adolescent overweight/obesity by a nonsignificant 17% (adjusted RR 0.83, 95% CI 0.49–1.43) and BMI by a nonsignificant −0.03 kg/m^2^ (95% CI −0.06 to 0.01).

In most cases, reducing family or neighbourhood disadvantage by only a small amount to average disadvantage (from quintile 1–2 to quintile 3), rather than to levels of least disadvantage (quintile 4–5), had minimal effects on either overweight/obese status or on BMI (Table 3).

#### Sensitivity analysis

Most effects remained similar when restricting the sample to those of European ancestry (Supplementary Table S6).

## Discussion

### Principal findings

This 12-year longitudinal study of approximately 1600 Australian children and 2000 adults showed a trend between increasing socioeconomic disadvantage, increased BMI, and probability of developing overweight or obesity. Children genetically predisposed to higher BMI were increasingly affected by socioeconomic disadvantage in family or neighbourhood settings. Post-hoc correlation analyses did not support neighbourhood “selection effects” (whereby families with higher polygenic risk scores might have come to live in certain neighbourhoods^36^) as the explanation for our findings.

Future work should confirm whether interventions targeting socioeconomic disadvantage^32^^,^^37^, ^38^, ^39^, ^40^, ^41^ could reduce high rates of obesity and shift population-level BMI distributions to healthier averages. Our hypothetical emulation of a target trial suggested that, at a population level (regardless of polygenic risk), interventions to improve children's neighbourhood and family disadvantage from the most to least disadvantage hold the potential to reduce the risk of adolescent overweight/obesity by 25% (neighbourhood intervention) and 39% (family intervention). Within this emulation, we show that effects are greater among vulnerable children with high polygenic risk: 32% risk reduction from neighbourhood intervention and 42% risk reduction from family-level intervention. However, our results suggest that reducing disadvantage by only a slight amount (i.e., to quintile 3) would have less risk reduction for the whole population and for children with high polygenic risk.

### Strengths and weaknesses of the study

Our population-derived cohort retained over 12 years spanned three important points of the life course and offers unique insight into obesity-related inequalities. However, as this was an observational study, recall bias or social desirability may have impacted the self-reported components of this study (e.g., parent-reported BMI or sociodemographics). Also, the genetic sub-sample represents only a subset (24–31%) of the original cohort, with the attrition over the 14 years since recruitment compounding LSAC's initial modest selection bias. Moreover, although our analytic sample was comparable to the full CheckPoint cohort on key demographics (e.g., disadvantage and BMI), we acknowledge that selection bias remains possible. We also acknowledge that reporting spatiotemporal effects was beyond the scope of this paper but recommend further research to examine relevant effects. Additionally, the adult CheckPoint cohort consists solely of parents (mostly mothers), further differentiating it from the overall adult population. It is a strength that LSAC has retained families with a wide range of social circumstances, but attrition within LSAC has been higher in disadvantaged families. Therefore, although commonly utilised in previous research,^19^ we acknowledge that using cohort-specific disadvantage quintiles may not be completely representative of Australian families living in the most disadvantaged circumstances. Although we measure both family and neighbourhood disadvantage, these are imperfect proxy measures of true disadvantage and these tools do not capture all aspects of socioeconomic disadvantage or other sources of marginalisation. The limited diversity in published genome-wide association studies limits generalisability of the results mostly to individuals of European genetic ancestry. Our best currently-available PRS does not fully represent the genetic contribution to obesity, accounting for a relatively modest amount of the variance (12% in children, 9% in adults) in BMI^22^ despite estimates of heritability being higher.^3^ Nonetheless, taking these limitations together, we believe that our findings are a likely underestimate of the strength of the relationship between socioeconomic disadvantage and polygenic risk for BMI.

### Strengths and weaknesses in relation to other studies

Trials to prevent or reduce childhood obesity have mostly focused on individual behaviour change, have been largely unsuccessful in achieving more than very small short-lived adiposity benefits,^42^ are mostly small in size,^43^ and in general have not considered genetic risk as an effect modifier. Thus, our hypothetical target trial explores hypotheses that to date have been impossible to test experimentally. Overall, although smaller than other cohorts, we extend previous obesity research into disadvantage and polygenic risk^8^^,^^10^^,^^11^^,^^13^ by examining multiple points across the life course in parallel population-based cohorts of children and adults with two distinct measures of socioeconomic disadvantage (family and neighbourhood). Among children, we define causal effects using the well-known target trial framework,^31^ whose public health implications lie in its ability to estimate real-world intervention effects.^28^ Although the possibility of bias or residual confounding (e.g., unmeasured confounders such as structural-level determinants of disadvantage and obesity) remains, this causal inference framework allows for tight adjustment for confounding and other biases which enhances the interpretability of findings.^28^ Implications are strengthened by examining both family and neighbourhood disadvantage to better capture multiple aspects of the obesogenic environment responsible for accentuating polygenic risk of obesity.^8^ This is an important point of difference with previous studies,^9^ as isolated targets (e.g., sugar-sweetened beverage intake) show limited evidence for directional and long-term associations with obesity in real-life cohorts.^44^

### Meaning of the study

For children with higher polygenic risk, the association between family or neighbourhood disadvantage and presentation of overweight or obesity is stronger than for those with lower polygenic risk. Socioeconomic disadvantage can increase the risk of inflammation, overweight, and later disease,^4^, ^5^, ^6^ which may result in deprivation amplification in families suffering both household-level and area-level disadvantage.^19^ These processes may be exacerbated among those with polygenic vulnerabilities to inflammatory diseases such as obesity.^8^^,^^11^^,^^13^ Socioeconomic disadvantage has downstream effects on obesogenic adversities such as poor health literacy, unhealthy diet, social isolation, and stress.^7^ Among adults, lifestyle and drug-based intervention can partially offset genetic risk for obesity,^45^^,^^46^ with those at high genetic risk potentially benefitting the most from population-level interventions.^46^ There are as yet no analogous data in children.

If living in disadvantage magnifies obesity risk among at-risk groups (e.g., children with genetic vulnerabilities), this provides further reason for direct intervention on disadvantage–in addition to equity being a basic human right. Meaningful impact on complex problems like adolescent obesity require multisectoral solutions beyond the health system.^2^ Factors like neighbourhood services, parent income, educational and employment opportunities are fundamental upstream determinants of health, and among the most readily available policy levers that governments hold. A reduction in obesity would almost certainly subsequently decrease associated non-communicable disease inequalities.

### Future research direction

Research efforts need to continue piloting system-wide programmes and policies to improve neighbourhood deprivation and family hardship (e.g., subsidised supermarkets, incentives to return to work). Governments can look towards new approaches to economic development such as community wealth building programmes targeted to disadvantaged neighbourhoods. In a natural experiment Rose et al. have recently demonstrated that such a programme (e.g., maximising socially productive use of land and property, investing in local wealth and supply changes, improving community employment conditions) can trigger community-level economic improvements that translate into better individual-level wellbeing.^38^ While neighbourhood interventions^39^ are generally acceptable to governments, communities, and families, future research should assess the acceptability and feasibility of individual-level socioeconomic intervention and their long-term impacts on obesity.

### Conclusion

Across the life course, living in greater socioeconomic disadvantage can be associated with higher risk for obesity, and often this association becomes more obvious for children and adults with high polygenic risk. Moreover, socioeconomic disadvantage disproportionately impacts the risk of obesity in children at higher polygenic risk of obesity. We recommend that future research and policy examine whether this double inequity could be reduced via societal efforts to reduce disadvantage as well as obesity, which could have life course benefits in population morbidity, mortality, and social capital.

## Contributors

JA Kerr, R Saffery, L Thornton, and M Wake were responsible for the concept and design of this study. JA Kerr and M Wake prepared the initial draft manuscript, and M Wake is the supervising author. Advised by M Downes, data analysis was conducted by JA Kerr, D Dumuid, and T Stanford. JA Kerr and K Lange directly accessed and verified the underlying data reported in the study. M Wake, R Saffery, D Burgner, J O'Sullivan, JA Kerr, K Lycett, TS Olds, B Edwards acquired study funding, or were responsible for project administration and resources. All authors contributed to the data interpretation and review, editing, and manuscript revisions. All authors, external and internal, had full access to the data (statistical reports and tables) in the study and are responsible for reported content and approve the manuscript as submitted.

## Data sharing statement

The publicly available LSAC and CheckPoint datasets generated and analysed during this study are available from the Australian National Centre for Longitudinal Data (dataverse.ada.edu.au/dataverse/lsac). R code for the Aim 1 GLMM models and bootstrapped confidence intervals are available at https://github.com/tystan/sociopolygenic. CheckPoint questionnaires, standard operating procedures, and data user guides are available at https://www.mcri.edu.au/research/projects/about-the-child-health-checkpoint. LSAC questionnaires, protocols, and data user guides are available at https://growingupinaustralia.gov.au/data-and-documentation.

## Declaration of interests

D Burgner (organising committee of the 2022 Excellence in Paediatrics Conference) and J O'Sullivan (Society for General Microbiology; 2022 Annual General Meeting) declare support for attending meetings. The author group declares no competing interests. All investigator funding is stated in the funding statement.
