# Supplementary material for: Socioeconomic disadvantage and polygenic risk of overweight in early and mid-life: a longitudinal population cohort study spanning 12 years
Source: Lancet Reg Health West Pac. 2024 Nov 13;53:101231. doi: 10.1016/j.lanwpc.2024.101231 (PMC11609315; doi:10.1016/j.lanwpc.2024.101231)
Supplement: Supplementary Figures and Tables [file mmc1.docx]

**Supplementary tables and figures for JA Kerr et al.**

**Socioeconomic disadvantage amplifies and risk of overweight in early and mid-life: A longitudinal population cohort study spanning 12 years**

**Supplementary Figure 1**: Association of disadvantage with BMI across childhood (page 3)

**Supplementary Figure 2:** BMI across childhood by family disadvantage (SEP) quintile, stratified by PRS quintile (page 4)

**Supplementary Figure 3:** Association of disadvantage with overweight/obese probability across childhood (page 5)

**Supplementary Figure 4:** Estimated probability of overweight/obesity across childhood by neighbourhood disadvantage (SEIFA) quintile, stratified by PRS quintile (page 6)

**Supplementary Figure 5:** Estimated probability of overweight/obesity across childhood by family disadvantage (SEP) quintile, stratified by PRS quintile (page 7)

**Supplementary Figure 6**: Association of disadvantage with BMI across adulthood (page 8)

**Supplementary Figure 7:** BMI across adulthood by family disadvantage (SEP) quintile, stratified by PRS quintile (page 9)

**Supplementary Figure 8:** Association of disadvantage with overweight/obese probability across adulthood (page 10)

**Supplementary Figure 9:** Overweight/obese probability across adulthood by neighbourhood disadvantage (SEIFA) quintile, stratified by PRS quintile (page 11)

**Supplementary Figure 10**: Overweight/obese probability across adulthood by family disadvantage (SEP) quintile, stratified by PRS quintile (page 12)

**Supplementary Figure 11:** Directed Acyclic Graph for Aim 2, example specific to target trial for neighbourhood socioeconomic disadvantage in early childhood (page 13)

**Supplementary Table 1**: The target trial and proposed emulation (page 14-15)

**Supplementary Table 2**: Description of Aim 2 co-variates (page 16)

**Supplementary Table 3:** Key characteristics of full CheckPoint Cohort by LSAC wave (page 17)

**Supplementary Table 4:** Estimated causal effect of childhood disadvantage on adolescent BMI and overweight/obesity adjusted for potential confounders, not stratified by polygenic risk score (page 18)

**Supplementary Table 5:** Percentage of children in disadvantage quintiles with overweight or obesity by adolescence (page 19)

**Supplementary Table 6:** Estimated causal effect of childhood disadvantage on adolescent BMI and overweight/obesity adjusted for potential confounders, limited to children of European descent (page 20)

**Supplementary Table 7:** Estimated BMI and 95% CI used to generate Main Figure 2; BMI across childhood by neighbourhood disadvantage (SEIFA) quintile, stratified by PRS quintile (page 21)

**Supplementary Table 8:** Estimated BMI and 95% CI used to generate Supplementary Figure 2; BMI across childhood by family disadvantage (SEP) quintile, stratified by PRS quintile (page 22)

**Supplementary Table 9:** Estimated BMI and 95% CI used to generate Main Figure 3; BMI across adulthood by neighbourhood disadvantage (SEIFA) quintile, stratified by PRS quintile (page 23)

**Supplementary Table 10:** Estimated BMI and 95% CI used to generate Supplementary Figure 7; BMI across adulthood by family disadvantage (SEP) quintile, stratified by PRS quintile (page 24)

**Supplementary Table 11:** Estimated probability of overweight/obesity (95% CI) used to generate Supplementary Figure 4; overweight/obesity probability across childhood by neighbourhood disadvantage (SEIFA) quintile, stratified by PRS quintile (page 25)

**Supplementary Table 12:** Estimated probability of overweight/obesity (95% CI) used to generate Supplementary Figure 5; overweight/obesity probability across childhood by family disadvantage (SEP) quintile, stratified by PRS quintile (page 26)

**Supplementary Table 13:** Estimated probability of overweight/obesity (95% CI) used to generate Supplementary Figure 9; overweight/obesity probability across adulthood by neighbourhood disadvantage (SEIFA) quintile, stratified by PRS quintile (page 27)

**Supplementary Table 14:** Estimated probability of overweight/obesity (95% CI) used to generate Supplementary Figure 10; overweight/obesity probability across adulthood by family disadvantage (SEP) quintile, stratified by PRS quintile (page 28)

**Supplementary Figure 1 Panel A: Association of SEIFA neighbourhood disadvantage with BMI (95% CI) across childhood; Panel B: Association of SEP family disadvantage with BMI (95% CI) across childhood; See Supplementary Figure 3 for overweight/obese proportions, rather than mean BMI. *In all cases the red quintile 1 represents the most disadvantage*.**


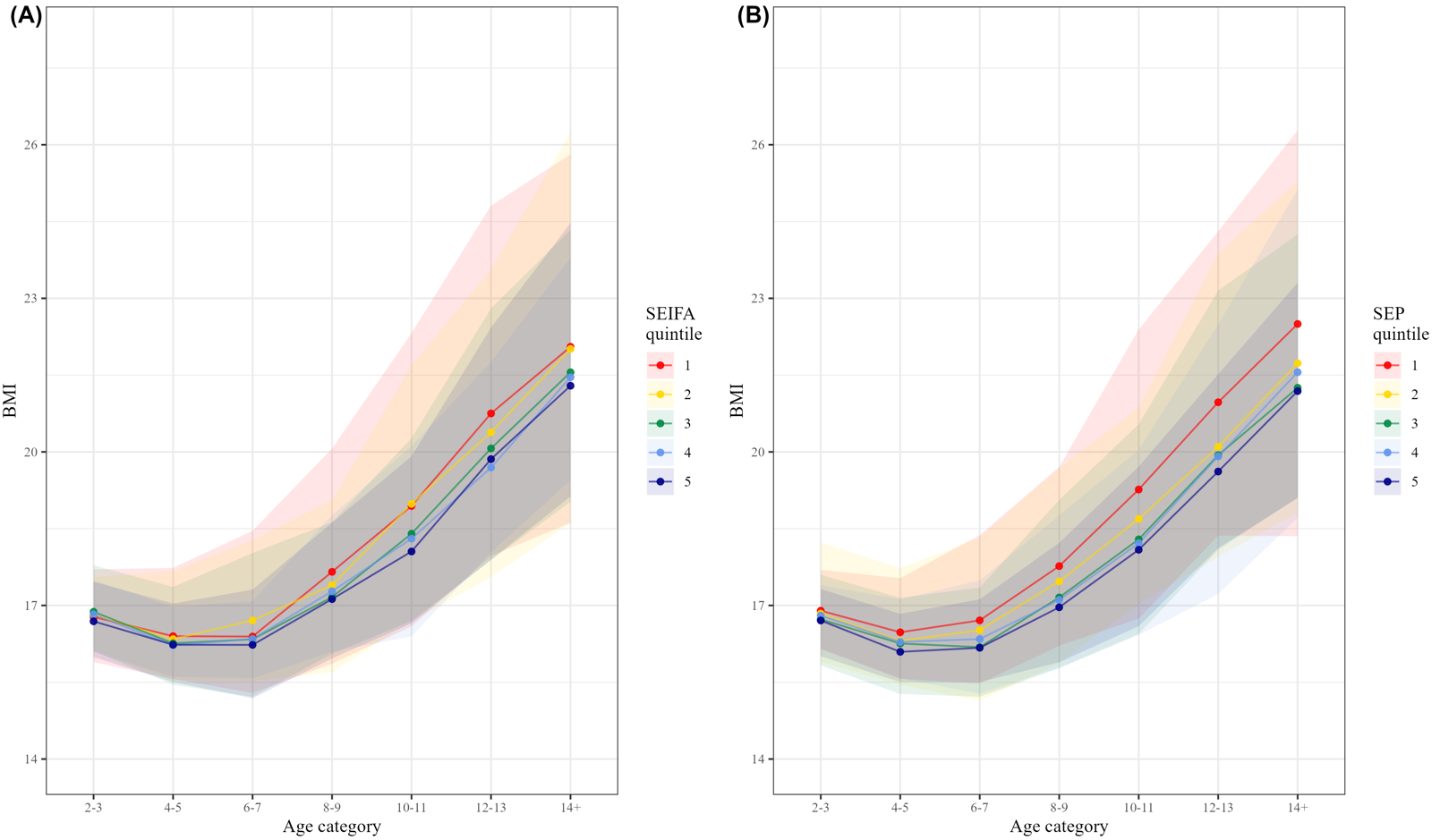


**Supplementary Figure 2: BMI across childhood by family disadvantage (SEP) quintile, stratified by PRS quintile**.


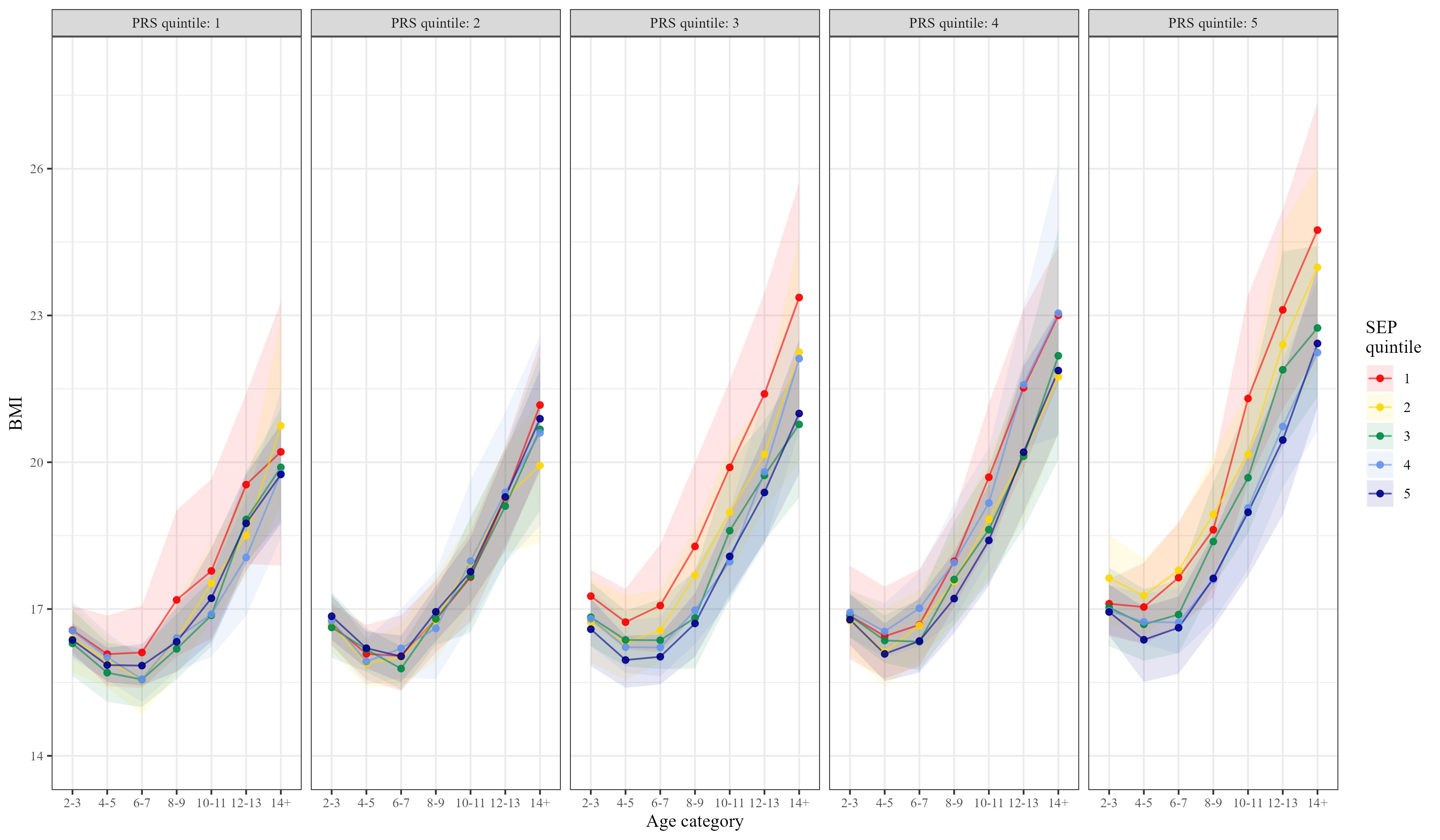


**Supplementary Figure 3 Panel A: Association of SEIFA neighbourhood disadvantage with overweight/obese probability (95% CI) across childhood; Panel B: Association of SEP family disadvantage with overweight/obese probability (95% CI) across childhood. *In all cases the red quintile 1 represents the most disadvantage*.**

**
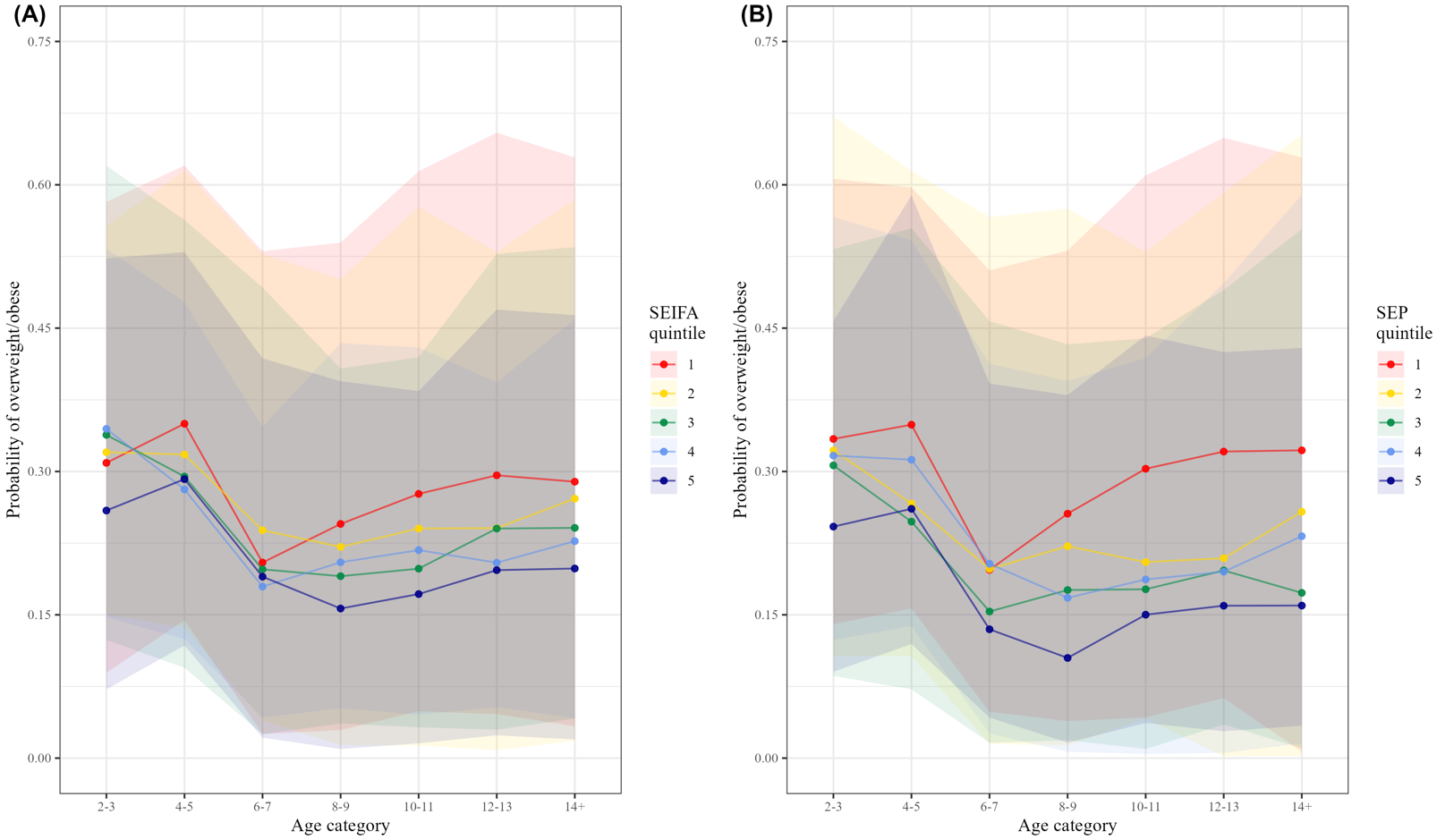
**

**Supplementary Figure 4: Estimated probability of overweight/obesity (95% CI) across childhood by neighbourhood disadvantage (SEIFA) quintile (1=most, 5=least disadvantage), stratified by PRS quintile (1=lowest, 5=highest risk).**

**
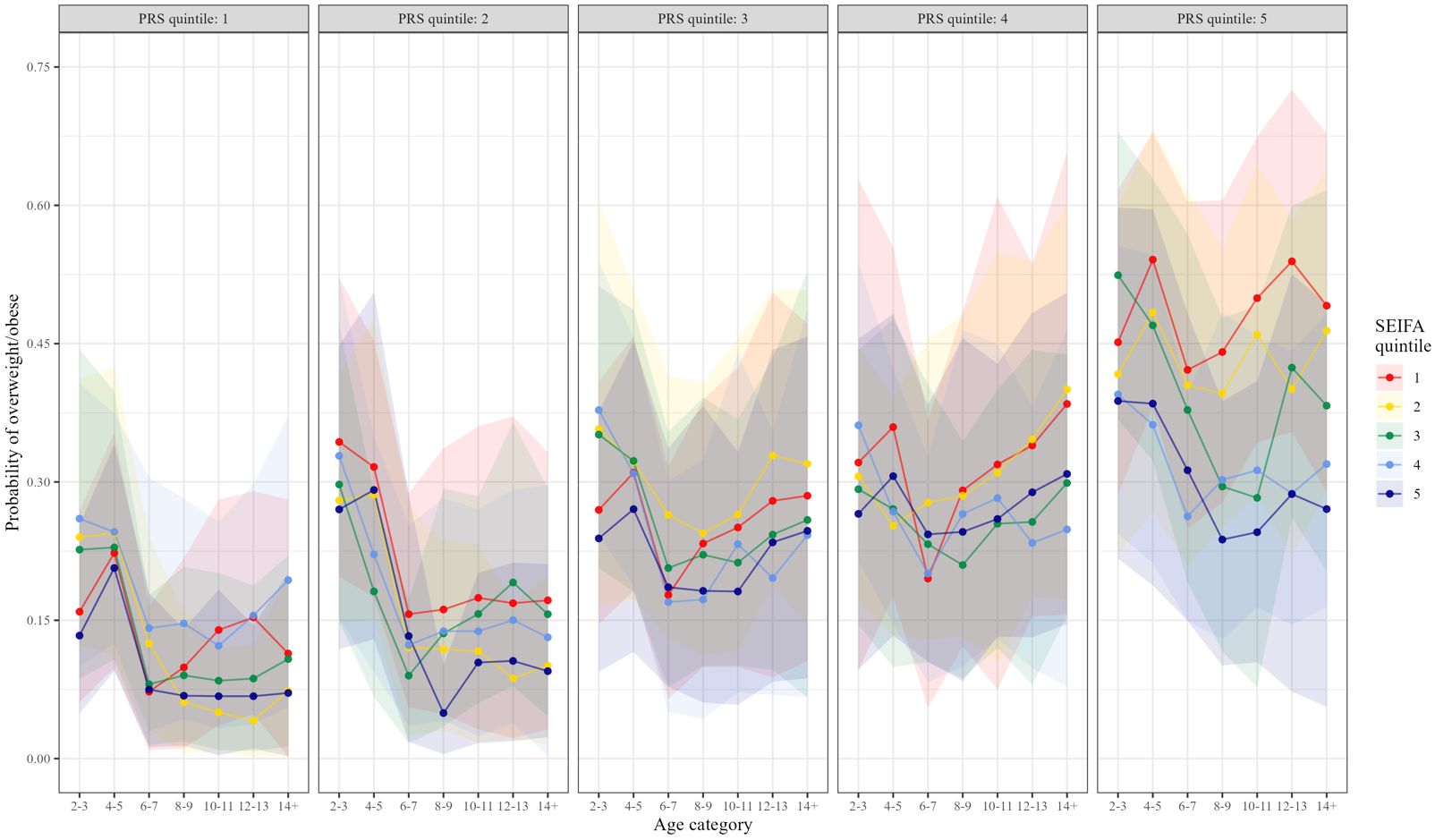
**

**Supplementary Figure 5: Estimated probability of overweight/obesity (95% CI) across childhood by family disadvantage (SEP) quintile (1=most, 5=least disadvantage), stratified by PRS quintile (1=lowest, 5=highest risk).**

**
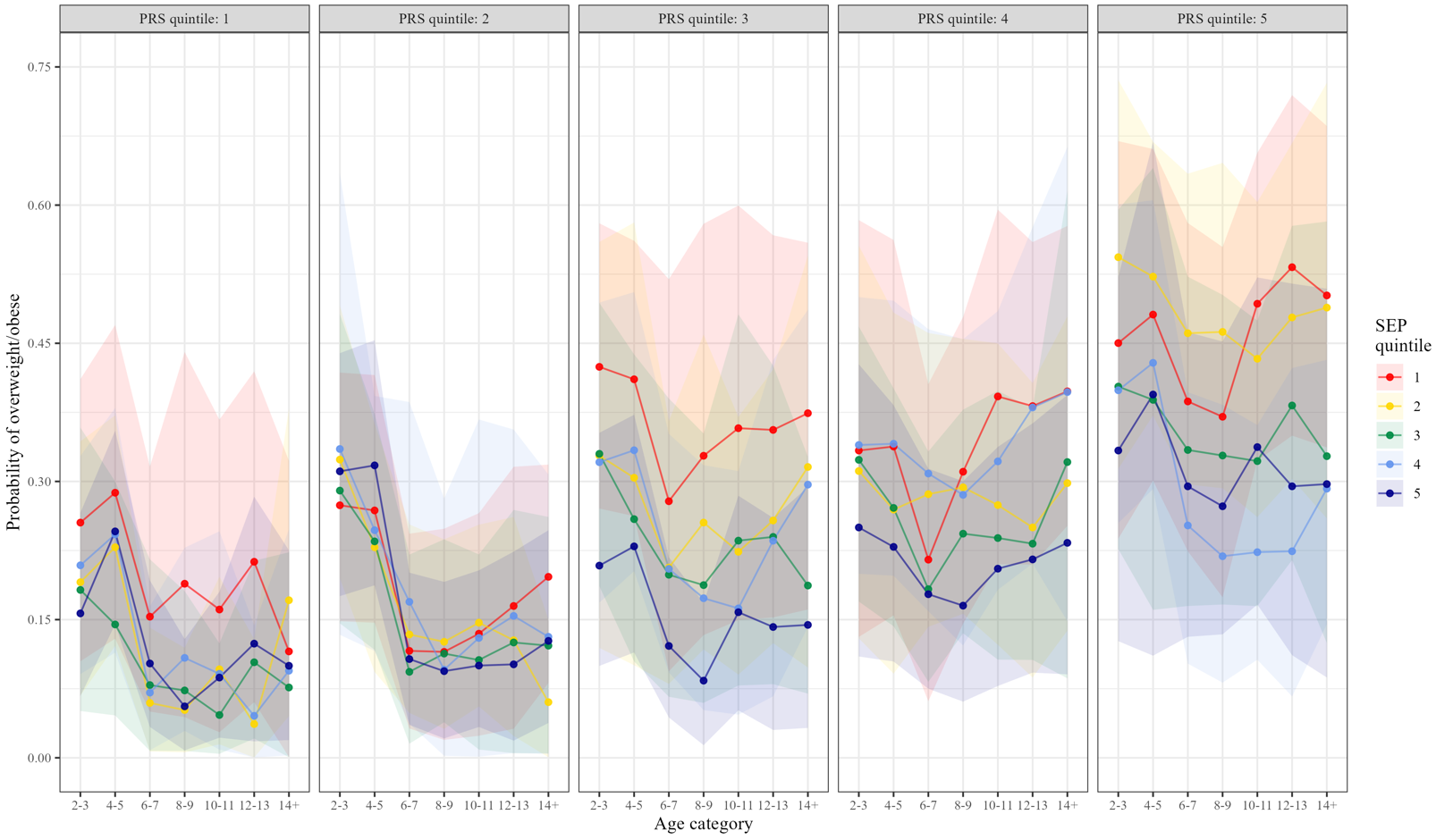
**

**Supplementary Figure 6 Panel A: Association of SEIFA neighbourhood disadvantage with BMI (95% CI) across adulthood; Panel B: Association of SEP family disadvantage with BMI (95% CI) across adulthood. *In all cases the red quintile 1 represents the most disadvantage*.**

**
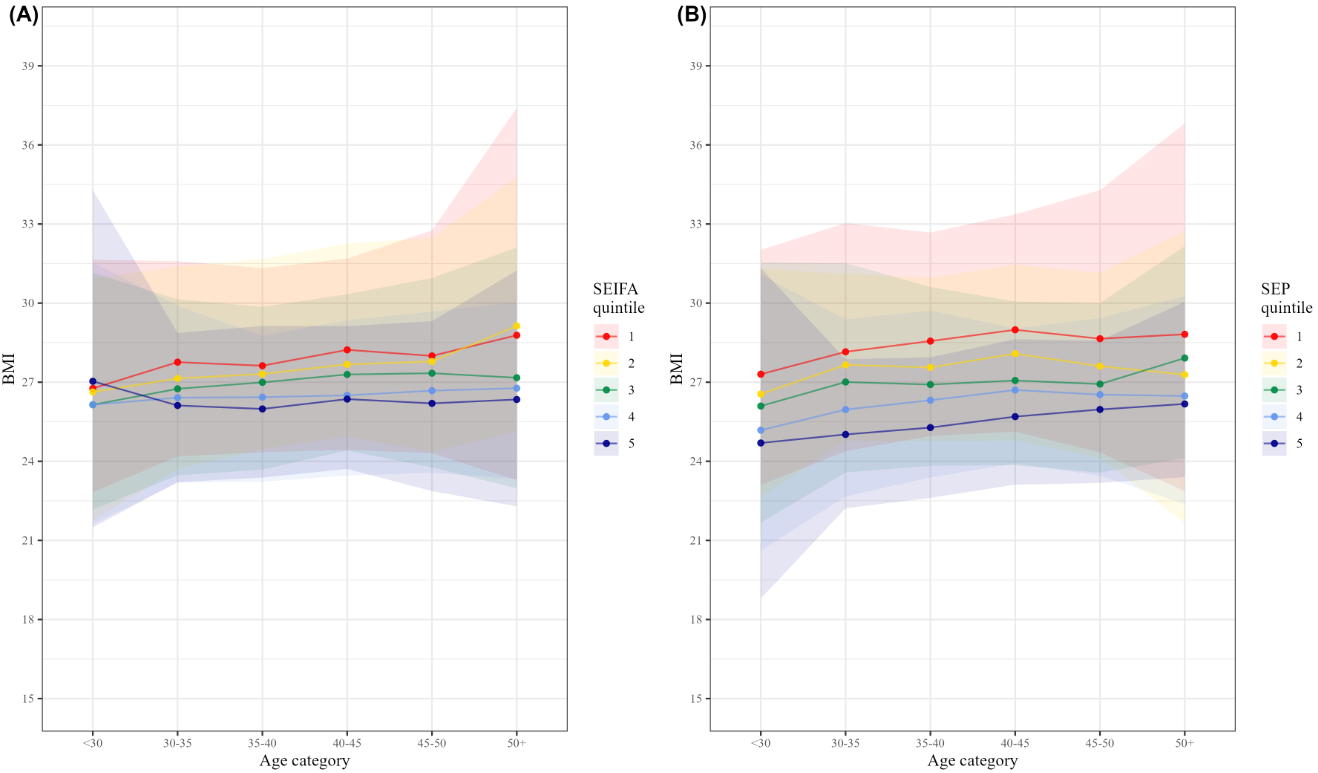
**

**Supplementary Figure 7:** BMI across adulthood by family disadvantage (SEP) quintile, stratified by PRS quintile.


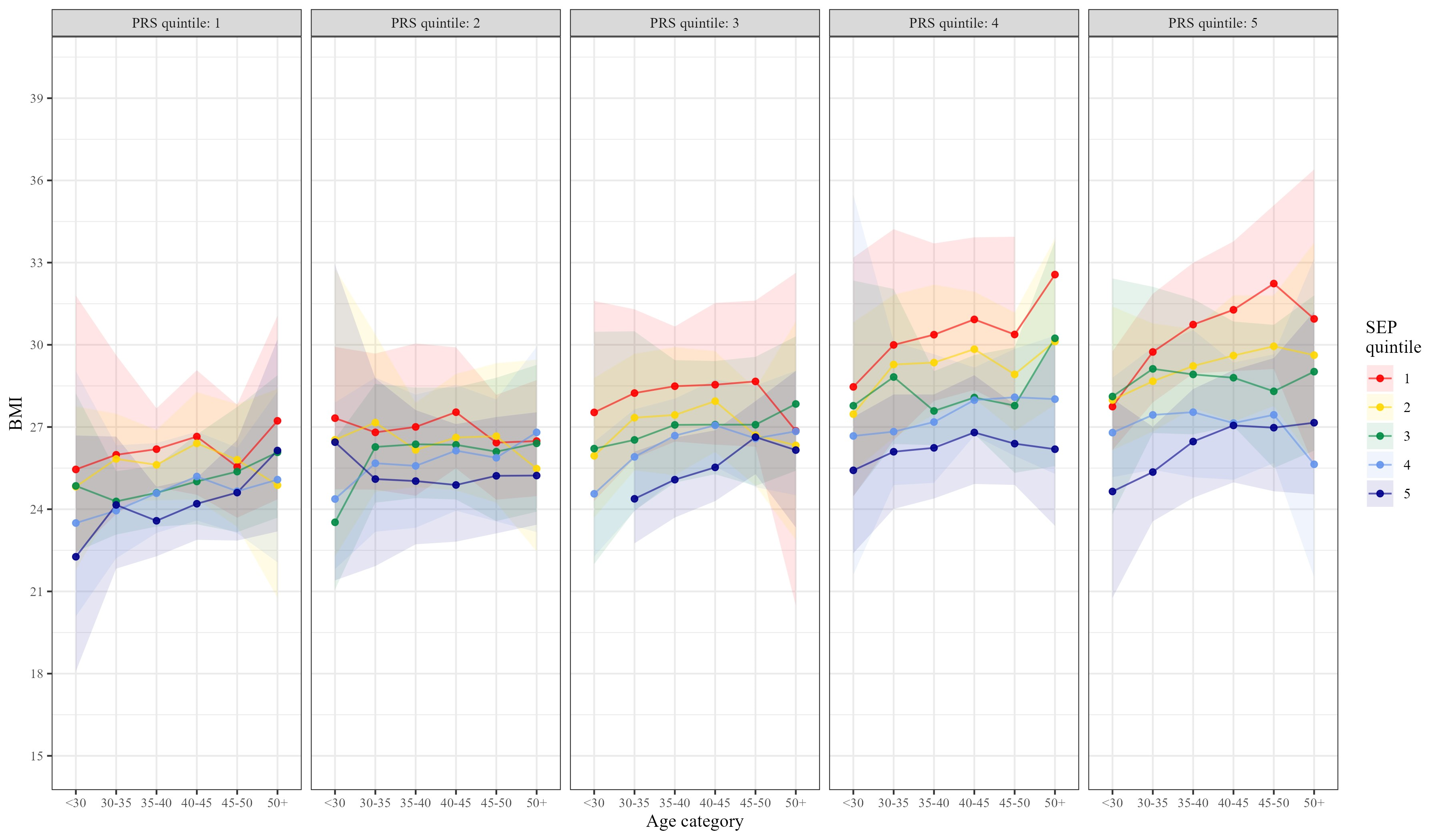


**Supplementary Figure 8: Panel A: Association of SEIFA neighbourhood disadvantage with overweight/obese probability (95% CI) across adulthood; Panel B: Association of SEP family disadvantage with overweight/obese probability (95% CI) across adulthood; *In all cases the red quintile 1 represents the most disadvantage*.**


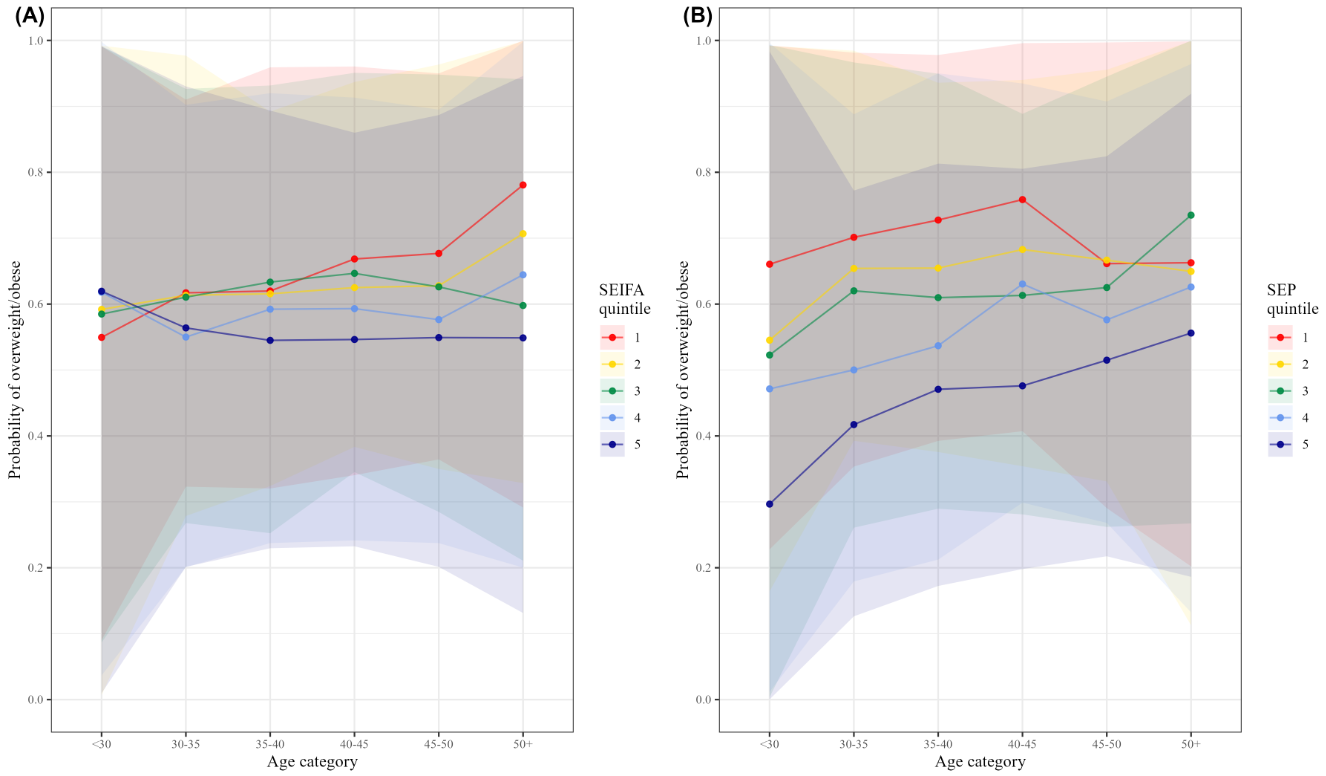


**Supplementary Figure 9: Estimated probability of overweight/obesity (95% CI) across adulthood by neighbourhood disadvantage (SEIFA) quintile (1=most, 5=least disadvantage), stratified by PRS quintile (1=lowest, 5=highest risk).**

**
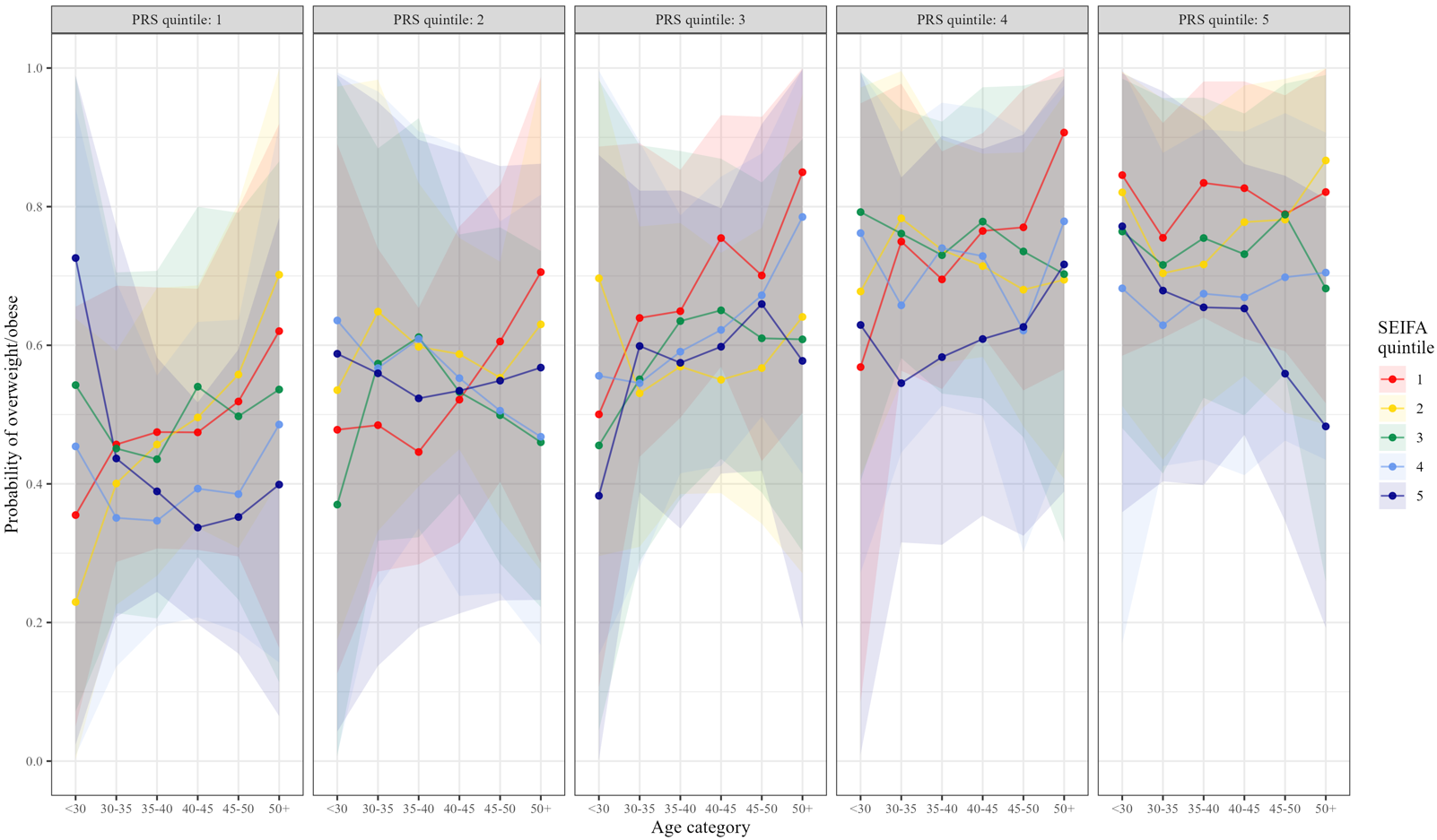
**

**Supplementary Figure 10: Estimated probability of overweight/obesity (95% CI) across adulthood by family disadvantage (SEP) quintile (1=most, 5=least disadvantage), stratified by PRS quintile (1=lowest, 5=highest risk).**

**
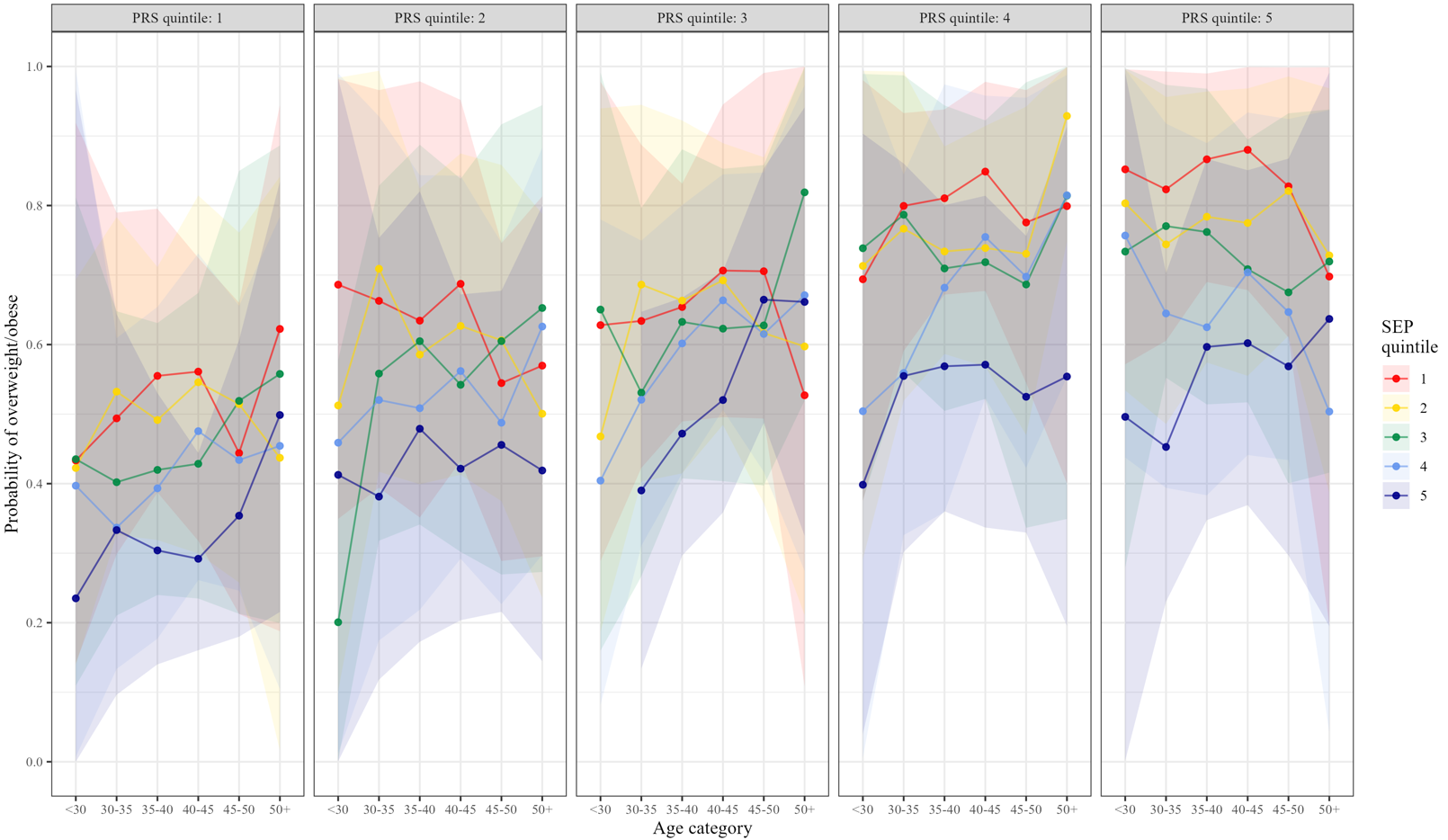
**

**Supplementary Figure 11: Directed Acyclic Graph for Aim 2, example specific to target trial for neighbourhood socioeconomic disadvantage in early childhood**

**
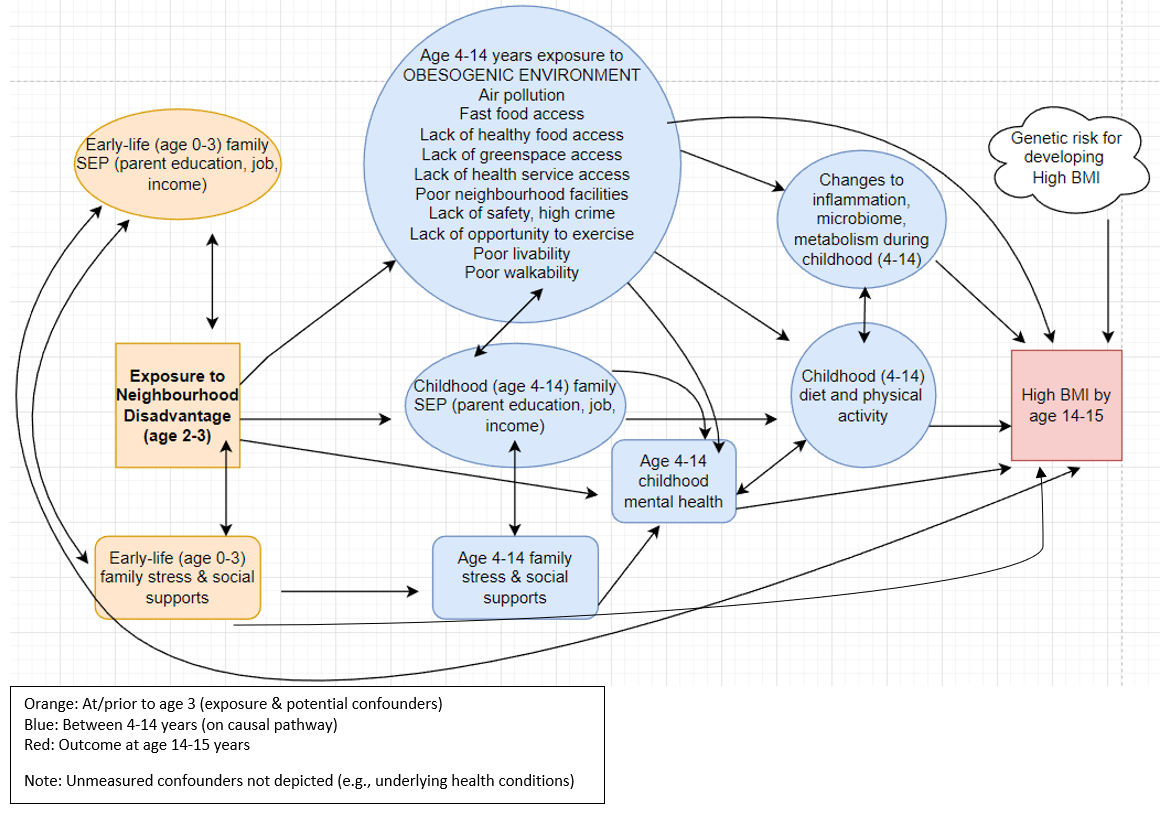
**

**Supplementary Table 1: The target trial and proposed emulation using LSAC B-cohort to estimate the causal effect of hypothetical intervention to reduce neighbourhood-level or family-level socioeconomic disadvantage (at 2-3 years or at 12-13 years) on adolescent obesity risk (14-15 years) in children of lower and higher polygenic risk for obesity.**

| **Protocol component** | **Target trial** | **Trial emulation** |
| --- | --- | --- |
| **Eligibility criteria** | **Target population:** Australian infants at birth in 2004  **Inclusion criteria:** Infants born in Australia during the year 2004.  **Exclusion criteria**: Lack of consent to providing a genetic sample. | **Analytic sample selection:** All B-cohort LSAC study participants comprised of a population-representative sample of all Australian infants aged 0-1 year in 2004 (N=5107; recruitment rate=57%). Cohort recruited through a two-stage cluster sampling method of the comprehensive Medicare database across Australia in March-November 2004.  Because subgroup analysis required data on polygenic risk for obesity, this cohort was then limited to those retained in LSAC until age 11-12 years, who consented and provided genetic data at the CheckPoint assessment wave (LSAC Wave 6.5).  **Approach to handling missing data and other potential sources of selection bias:** Thereafter, because missing outcome data was minimal, all participants were retained in the sample if they had provided at least one measurement of body mass index (BMI) between ages 2-3 and ages 14-15. Missingness on key variables BMI and disadvantage was <6% and the analytic sample was comparable to the CheckPoint cohort on key demographics. Therefore, available case analysis (range n=1056 to 1250) was conducted and multiple imputation was not employed. |
| **Treatment strategies** | **Treatment arms in the trial:**  **Intervention 1:** Exposure to low (the least) levels of family-level or neighbourhood-level disadvantage.  **Intervention 2:** Exposure to average levels of family-level or neighbourhood-level disadvantage.  **Comparator:** Exposure to high (the most) levels of family-level or neighbourhood-level disadvantage.  Real-world examples of interventions to improve family-level disadvantage could include reducing barriers to accessing health services, healthy and safe housing, education, and employment by addressing hardship and structural obstacles through measures like job creation, educational engagement, and increasing household income (e.g., tax benefits or cash transfers; incentives for parents to return to workforce).  Real-world examples of intervention to improve neighbourhood disadvantage could include community wealth building programmes, targeted area-level education/employment initiatives, supported community garden initiatives, targeted areal-level and equitably distributed health/social services, healthy urban planning requirements (e.g., placement of greenspace, footpaths, fast food outlets, local fresh food markets), targeted area-level healthy home requirements, and subsidised supermarkets and sports/recreational centres. | **Treatment exposure measure at child age 2-3 years (wave 2) and child age 12-13 years (wave 7):**  ***Neighbourhood-Level Intervention***  **Neighbourhood Intervention 1 “Low Disadvantage”:** Defined as being quintile 4 or 5 neighbourhood disadvantage (SEIFA IRSD score).  **Neighbourhood Intervention 2 “Average Disadvantage”:** Defined as being quintile 3 neighbourhood disadvantage (SEIFA IRSD score).  **Comparator “High Levels of Neighbourhood Disadvantage”:** Defined as being quintile 1 or 2 neighbourhood disadvantage (SEIFA IRSD score).  ***Family-Level Intervention***  **Family-Level Intervention 1 “Low Disadvantage”:** Defined as being quintile 4 or 5 family/household socioeconomic position (SEP).  **Family-Level Intervention 2 “Average Disadvantage”:** Defined as being quintile 3 family/household socioeconomic position (SEP).  **Comparator “High Levels of Family Socioeconomic Disadvantage”:** Defined as being quintile 1 or 2 family/household socioeconomic position (SEP). |
| **Assignment procedures** | **Randomisation strategy:**  Randomisation at recruitment (assignment non-blinded) | **Selection of confounders (exposure: neighbourhood disadvantage):**  Family-level disadvantage at the LSAC wave prior exposure time point  Sex and age  Genetic population structure and DNA sample type  Family/household conflict at the LSAC wave prior exposure time point  Parental mental health at the LSAC wave prior exposure time point  **Approach to adjustment:** Outcome regression |
| **Follow-up period** | **Start:** Wave 1 recruitment and randomisation.  (birth, baseline time zero)  **Ends:** Wave 8 child age 14-15 years. | **Timing of measures:**  **Starts:** At birth, child age 0-1 year  **Ends:** At adolescence, child age 14-15 years |
| **Outcome** | **Outcomes:**  Body Mass Index (BMI)  Binary Overweight or Obesity status | **Outcome measures at age 14-15 years:**  Raw BMI calculated as weight(kg)/(height(m)^2^)  Centers for Disease Control (CDC) growth reference values applied to BMI to determine adolescent overweight/obesity status at the ≥85th percentile. |
| **Subgroup analyses** | **Subgroups of the population for which it is of interest to obtain separate effects:**  Lower vs. higher polygenic risk score (PRS) for BMI | **Subgroup variable measures and approach:**  **Measure:** Median split of PRS in analytic sample.  **Approach:** Stratification in regression. Separate regression models generated for children with high and low PRS. |
| **Causal contrast of interest** | Mean difference in BMI between intervention and comparator arms in the target population.  Risk of overweight/obesity in each intervention arm relative to the comparator arm in the target population |  |

**Supplementary Table 2: Description of Aim 2 covariates**

| **Construct** | **Measure** | **Collection** | | **Brief protocol** |
| --- | --- | --- | --- | --- |
|  | | **LSAC (Waves 1-8)** | **Check Point (Wave 6·5)** |  |
| Population structure | Genetic principal components |  | ● | Because the PRS was created with summary data from European populations,^1^ we adjust for population structure to account for any systematic differences between different genetic sub-populations within the cohort, such as differences in allele frequencies that can contribute to the phenotype in different ways.^2^ To make this adjustment, models were adjusted for the top five principal components from a Principal Components Analysis of the CheckPoint genetics dataset. These components represent the largest variations in allele frequencies (i.e., different genetic sub-populations) within the cohort.^3^ |
| DNA sample type | Bloods, saliva |  | ● | Primary DNA sample type (venous blood, dried blood spot, or oral) was adjusted for in the models, because genotype quality can be subject to sample quality, and because sample type differed by type of CheckPoint assessment (main centre vs home visit). |
| Earlier Disadvantage | Neighbourhood and Family | ● |  | Measured as described in Table 1, except taken from the LSAC wave preceding the Aim 2 exposure time point (e.g., Wave 1 SEP for models with Wave 2 (age 2-3 years) SEIFA as the exposure). |
| Family conflict | Mother-rated argumentative parental relationships & hostile parenting | ● |  | Family conflict preceding the exposure time point was measured with a 3-item argumentative relationship scale adapted from the Quality of Co-parental Interaction Scale.^4^ At the wave preceding the exposure time point, mothers self-reported on a 5-point Likert scale from 1 “never” to 5 “always” how often: is there anger or hostility between you and your partner, is your conversation awkward or stressful, do you and your partner disagree about basic child-rearing issues. The mean was generated to indicate conflict level.  Adapted from measures of hostile parenting,^5-6^ at Wave 1 hostile parenting preceding the Wave 2 exposure time point (i.e., child age 2-3 years) was measured with a 3-item scale. Mother’s self-reported on a 10-point Likert scale from 1 “not at all” to 10 “all the time” in the last 4-weeks I have: been angry with this child, raised my voice or shouted at this child, lost my temper with this child. The mean was generated to indicate degree of hostile parenting.  At Wave 6 hostile parenting preceding the Wave 7 exposure time point (i.e., child age 12-13 years) was measured with a 6-item angry parenting scale.^7^ Mother’s self-reported behaviours on a 5-point Likert scale from 1 “never/almost never” to 5 “all the time”. Example items include, how often do you think that the level of punishment you give this child depends on your mood, or how often are you angry when you punish this child? The mean was generated to indicate degree of hostile/angry parenting. |
| Parental mental health | Parental Psychological Distress Scale | ● |  | Parental mental health preceding the exposure time point was measured with mother and father self-reported distress using the Kessler Psychological Distress Scale (K6).^8^ This tool estimates serious non-specific mental illness at the population level, with participants rating six items on a 5-point Likert scale from 1 “all of the time” to 5 “none of the time”, items are then summed. Examples include “in the past 4 weeks about how often did you feel: nervous, or so sad that nothing could cheer you up.” |
| Abbreviations: LSAC: Longitudinal Study of Australian Children; SEP: socio-economic position; SEIFA: Socio-Economic Index for Areas.  References  1: Khera AV, Chaffin M, Wade KH, et al. Polygenic prediction of weight and obesity trajectories from birth to Adulthood· *Cell* 2019;177:587-96.  2: Choi SW, Mak TSH, O’Reilly PF. Tutorial: a guide to performing polygenic risk score analyses. *Nature Protocols* 2020;15: 2759–72.  3: Lange K, Kerr JA, Mansell T, et al. Can adult polygenic scores improve prediction of body mass index in childhood? *Int J Obesity* 2022;46:1375-83.  4: Ahrons CR. The continuing co-parental relationship between divorced spouses. *AJO* 1981;51:415-28·  5: U S Department of Education. Early Childhood Longitudinal Study, Birth Cohort: National Centre for Education Statistics. 2001.  6: Statistics Canada. National Longitudinal Survey of Children and Youth (NLSCY) Cycle 3 survey instruments: parent questionnaire. Ottowa, Canada. 2000a.  7: Chao RKW, Willms JD. The effects of parenting practices on children’s outcomes. In D Lillms (Ed.)., Vulnerable Children. Edmonton. Alberta, Canada: University of Alberta Press. 2002.  8: Kessler RC, Andrews G, Colpe LJ, et al. Short screening scales to monitor population prevalences and trends in non‐specific psychological distress. *Psychol Med* 2002;32:959–76. | | | | |

**Supplementary Table 3: Key characteristics of full CheckPoint Cohort by LSAC wave (i.e., not limited to those with polygenic risk scores)**

|  |  | **Demographics** | |  | **Disadvantage** | |  | **Body Mass** | | |
| --- | --- | --- | --- | --- | --- | --- | --- | --- | --- | --- |
|  |  | **Age** | **Male %** |  | **Family SEP z-score** | **Area SEIFA score** |  | **BMI z-score** | **BMI** | **OVOB %** |
| **Children** |  |  |  |  |  |  |  |  |  |  |
| Wave 2 |  | 2·3 (0·4) | 51.0 |  | 0·27 (0·96) | 1021 (58) |  | 0·5 (1·1) | 16·8 (1·5) | 31.8 |
| Wave 3 |  | 4·2 (0·4) | 51.0 |  | 0·26 (0·95) | 1025 (58) |  | 0·5 (1·0) | 16·3 (1·6) | 31.1 |
| Wave 4 |  | 6·3 (0·5) | 51.0 |  | 0·23 (0·96) | 1024 (57) |  | 0·3 (1.0) | 16·4 (2·1) | 20.6 |
| Wave 5 |  | 8·4 (0·5) | 50·6 |  | 0·21 (0·99) | 1025 (66) |  | 0·3 (1.0) | 17·3 (2·6) | 20.3 |
| Wave 6 |  | 10·4 (0·5) | 51.1 |  | 0·17 (0·99) | 1027 (61) |  | 0.3 (1·0) | 18·6 (3·1) | 22.5 |
| Wave 7 |  | 12·4 (0·5) | 51.0 |  | 0·16 (0·99) | 1027 (63) |  | 0·2 (1·0) | 20·2 (3·6) | 23.8 |
| Wave 8 |  | 14·3 (0·5) | 50·6 |  | 0·14 (0·97) | 1028 (63) |  | 0·4 (1·0) | 21·7 (3·9) | 25.6 |
|  |  |  |  |  |  |  |  |  |  |  |
| **Adults** |  |  |  |  |  |  |  |  |  |  |
| Wave 2 |  | 35·4 (5·3) | 35.3 |  | 0·32 (0·94) | 1022 (58) |  | ·· | 25·9 (4·5) | 52.9 |
| Wave 3 |  | 37·4 (5·3) | 35.3 |  | 0·31 (0·94) | 1026 (58) |  | ·· | 26·2 (4·7) | 54.6 |
| Wave 4 |  | 39·4 (5·4) | 35.1 |  | 0·29 (0·95) | 1025 (57) |  | ·· | 26·7 (5·4) | 57.3 |
| Wave 5 |  | 41·4 (5·4) | 35.1 |  | 0·26 (0·98) | 1029 (60) |  | ·· | 26·9 (5·4) | 58.8 |
| Wave 6 |  | 43·3 (5·6) | 35.1 |  | 0·23 (0·97) | 1029 (60) |  | ·· | 27·2 (5·5) | 60.6 |
| Wave 7 |  | 45·4 (5·5) | 35.3 |  | 0·21 (0·97) | 1029 (62) |  | ·· | 27·7 (6·0) | 63.1 |
| Wave 8 |  | 47·3 (5·5) | 34.8 |  | 0·18 (0·96) | 1029 (63) |  | ·· | 27·8 (5.8) | 65.8 |

**Supplementary Table 4: Estimated causal effect of childhood disadvantage on adolescent BMI and overweight/obesity adjusted for potential confounders, not stratified by polygenic risk score.**

| **Outcome:** | **Overweight or Obesity at 14-15 years** | | | | |  | **Body Mass Index at 14-15 years** | | | | |
| --- | --- | --- | --- | --- | --- | --- | --- | --- | --- | --- | --- |
| **Exposure to neighbourhood disadvantage (SEIFA, IRSD) at:** | **Early Childhood ages 2-3** | |  | **Late Childhood ages 12-13** | |  | **Early Childhood ages 2-3** | |  | **Late Childhood ages 12-13** | |
|  | **RR (95% CI)** | **p** |  | **RR (95% CI)** | **p** |  | **MD (95% CI)** | **p** |  | **MD (95% CI)** | **p** |
| **Whole Cohort** | |  |  |  |  |  |  |  |  |  |  |
| **Compared to living in disadvantage** |  |  |  |  |  |  |  |  |  |  |  |
| Living in average disadvantage | 1.01 (0.75, 1.37) | .95 |  | 0.96 (0.69, 1.33) | .79 |  | 0.00 (-0.04, 0.04) | .89 |  | -0.01 (-0.05, 0.03) | .55 |
| Living in least disadvantage | 0.75 (0.57, 1.00) | .05 |  | 0.90 (0.66, 1.22) | .50 |  | -0.01 (-0.04, 0.02) | .60 |  | -0.02 (-0.05, 0.01) | .12 |
|  | | | | | | | | | | | |
| **Exposure to family disadvantage (SEP) at:** | **Early Childhood ages 2-3** | |  | **Late Childhood ages 12-13** | |  | **Early Childhood ages 2-3** | |  | **Late Childhood ages 12-13** | |
|  | **RR (95% CI)** | **p** |  | **RR (95% CI)** | **p** |  | **MD (95% CI)** | **p** |  | **MD (95% CI)** | **p** |
| **Whole Cohort** | |  |  |  |  |  |  |  |  |  |  |
| **Compared to living in disadvantage** |  |  |  |  |  |  |  |  |  |  |  |
| Living in average disadvantage | 0.77 (0.55, 1.08) | .13 |  | 0.74 (0.52, 1.01) | .09 |  | -0.04 (-0.08, -0.01) | .02 |  | -0.03 (-0.07, 0.00) | .06 |
| Living in least disadvantage | 0.61 (0.45, 0.82) | ·001 |  | 0.64 (0.48, 0.86) | ·003 |  | -0.05 (-0.08, -0.02) | .003 |  | -0.05 (-0.08, -0.01) | .01 |
| SEIFA-specific intervention models adjusted for sex, age, and confounders measured 2 years prior to the exposure: *family-level disadvantage* (SEP: parent income, occupation, education), family household conflict, parental mental health.  SEP-specific intervention models adjusted for sex, age, and confounders measured 2 years prior to the exposure: *neighbourhood-level disadvantage* (SEIFA, IRSD), family household conflict, parental mental health.  RR: risk ratio; MD: mean difference; SEIFA: Socio-Economic Indexes for Areas; IRSD: Index of Relative Socioeconomic Disadvantage; SEP: socioeconomic position; CI: confidence interval.  Disadvantage: Quintile 1-2; Average Disadvantage: Quintile 3; Least Disadvantage: Quintile 4-5.  Survey weighting applied to all analysis. | | | | | | | | | | | |

**Supplementary Table 5: Percentage of children in disadvantage quintiles with overweight or obesity by adolescence**

| **Exposure to neighbourhood disadvantage (SEIFA) at:** | **Exposure at ages 2-3** | **Exposure at ages 12-13** |
| --- | --- | --- |
|  | **% Overweight or Obese at 14-15 years** | |
| **High Polygenic Risk Score (PRS > median split)** | |  |
| Living in most disadvantage (Q1 & Q2) | 41% | 45% |
| Living in average disadvantage (Q3) | 44% | 31% |
| Living in least disadvantage (Q4 & Q5) | 27% | 27% |
| **Low Polygenic Risk Score (PRS < median split)** | |  |
| Living in most disadvantage (Q1 & Q2) | 18% | 16% |
| Living in average disadvantage (Q3) | 15% | 20% |
| Living in least disadvantage (Q4 & Q5) | 14% | 14% |
|  |  |  |
| **Exposure to family disadvantage (SEP) at:** | **Exposure at ages 2-3** | **Exposure at ages 12-13** |
|  | **% Overweight or Obese at 14-15 years** | |
| **High Polygenic Risk Score (PRS > median split)** | |  |
| Living in disadvantage (Q1 & Q2) | 46% | 44% |
| Living in average disadvantage (Q3) | 35% | 30% |
| Living in least disadvantage (Q4 & Q5) | 24% | 29% |
| **Low Polygenic Risk Score (PRS < median split)** | |  |
| Living in disadvantage (Q1 & Q2) | 19% | 18% |
| Living in average disadvantage (Q3) | 13% | 15% |
| Living in least disadvantage (Q4 & Q5) | 13% | 14% |
| Survey weighting applied | | |

**Supplementary Table 6: Estimated causal effect of childhood disadvantage on adolescent BMI and overweight/obesity adjusted for potential confounders, limited to children of European descent** **(n=1365)**

| **Outcome:** | | **Overweight or Obesity at 14-15 years** | | | | |  | **Body Mass Index at 14-15 years** | | | | |
| --- | --- | --- | --- | --- | --- | --- | --- | --- | --- | --- | --- | --- |
| **Intervention to improve neighbourhood disadvantage (SEIFA) at:** | | **Early Childhood ages 2-3** | |  | **Late Childhood ages 12-13** | |  | **Early Childhood ages 2-3** | |  | **Late Childhood ages 12-13** | |
|  | | **RR (95% CI)** | **p** |  | **RR (95% CI)** | **p** |  | **MD (95% CI)** | **p** |  | **MD (95% CI)** | **p** |
| **High Polygenic Risk Score (PRS)** | | |  |  |  |  |  |  |  |  |  |  |
| **Compared to living in disadvantage (Q1 & Q2)** | |  |  |  |  |  |  |  |  |  |  |  |
| Living in average disadvantage (Q3) | | 0·95 (0.68, 1.34) | .77 |  | 0.73 (0.48, 1.11) | .14 |  | -0.01 (-0.06, 0.04) | .73 |  | -0.04 (-0.10, 0.01) | .11 |
| Living in least disadvantage (Q4 & Q5) | | 0.62 (0.43, 0.89) | .01 |  | 0.70 (0.48, 1.03) | .07 |  | -0.02 (-0.07, 0.02) | .32 |  | -0.05 (-0.09, -0.00) | .03 |
| **Low Polygenic Risk Score (PRS)** | | |  |  |  |  |  |  |  |  |  |  |
| **Compared to living in disadvantage (Q1 & Q2)** | |  |  |  |  |  |  |  |  |  |  |  |
| Living in average disadvantage (Q3) | | 1.13 (0.65, 1.99) | .66 |  | 1.28 (0.73, 2.26) | .38 |  | 0.01 (-0.03, 0.05) | .63 |  | 0.01 (-0.04, 0.06) | .59 |
| Living in least disadvantage (Q4 & Q5) | | 1.03 (0.60, 1.79) | .91 |  | 1.45 (0.82. 2.58) | .20 |  | 0.00 (-0.04, 0.04) | .99 |  | 0.01 (-0.02, 0.05) | .44 |
|  |  | | | | | | | | | | | |
| **Intervention to improve family disadvantage (SEP) at:** | | **Early Childhood ages 2-3** | |  | **Late Childhood ages 12-13** | |  | **Early Childhood ages 2-3** | |  | **Late Childhood ages 12-13** | |
|  | | **RR (95% CI)** | **p** |  | **RR (95% CI)** | **p** |  | **MD (95% CI)** | **p** |  | **MD (95% CI)** | **p** |
| **High Polygenic Risk Score (PRS)** | | |  |  |  |  |  |  |  |  |  |  |
| **Compared to living in disadvantage (Q1 & Q2)** | |  |  |  |  |  |  |  |  |  |  |  |
| Living in average disadvantage (Q3) | | 0.83 (0.55, 1.25) | .37 |  | 0.81 (0.49, 1.33) | .40 |  | -0.04 (-0.09, 0.01) | .15 |  | -0.01 (-0.07, 0.04) | .66 |
| Living in least disadvantage (Q4 & Q5) | | 0·56 (0·40, 0·79)  0.61 (0.44, 0.85) | ·001  .003 |  | 0·71 (0·51, 0·98)  0.85 (0.61, 1.18) | ·04  .33 |  | -0·06 (-0·10, -0·02)  -0.05 (-0.10, -0.01) | ·01  .02 |  | -0·02 (-0·06, 0·02)  -0.00 (-0.05. 0.04) | ·27  .87 |
| **Low Polygenic Risk Score (PRS)** | | |  |  |  |  |  |  |  |  |  |  |
| **Compared to living in disadvantage (Q1 & Q2)** | |  |  |  |  |  |  |  |  |  |  |  |
| Living in average disadvantage (Q3) | | 0.90 (0.48, 1.70) | .74 |  | 1.01 (0.51, 2.00) | .99 |  | -0.02 (-0.06, 0.02) | .25 |  | -0.02 (-0.07, 0.03) | .52 |
| Living in least disadvantage (Q4 & Q5) | | 0.77 (0.45, 1.31) | .33 |  | 0.78 (0.48, 1.28) | .32 |  | -0.04 (-0.07, 0.00) | .07 |  | -0.04 (-0.08, -0.00) | .05 |
| SEIFA-specific intervention models adjusted for sex, age, genetic principal components, genetic collection/sample type, and confounders measured 2 years prior to the exposure: *family-level disadvantage* (SEP: parent income, occupation, education), family household conflict, parental mental health.  SEP-specific intervention models adjusted for sex, age, genetic principal components, genetic collection/sample type, and confounders measured 2 years prior to the exposure: *neighbourhood-level disadvantage* (SEIFA, IRSD), family household conflict, parental mental health.  High polygenic risk defined at median split. Survey weighting applied to all analysis. | | | | | | | | | | | | |

**Supplementary Table 7: Estimated BMI (95% CI in brackets) used to generate Main Figure 1; “Estimated BMI across childhood by neighbourhood disadvantage (SEIFA) quintile (1=most, 5=least disadvantage), stratified by PRS quintile (1=lowest, 5=highest risk)”.**

|  |  | **Age** | | | | | | |
| --- | --- | --- | --- | --- | --- | --- | --- | --- |
| **SEIFA** | **PRS** | **2-3y** | **4-5y** | **6-7y** | **8-9y** | **10-11y** | **12-13y** | **14y+** |
| 1 | 1 | 16·4 (15·7, 16·9) | 15·9 (15·4, 16·4) | 15·6 (15·1, 16·2) | 16·6 (15·6, 17·7) | 17·6 (16·6, 18·8) | 19·4 (18·1, 20·9) | 20·0 (18·3, 22·3) |
| 1 | 2 | 16·7 (16·2, 17·4) | 16·1 (15·5, 16·6) | 16·1 (15·5, 16·7) | 17·0 (15·8, 18·1) | 18·0 (16·2, 19·4) | 19·4 (17·6, 21·2) | 20·9 (18·7, 22·8) |
| 1 | 3 | 16·7 (16·2, 17·2) | 16·3 (15·8, 16·7) | 16·3 (15·7, 16·9) | 17·5 (16·5, 18·4) | 18·7 (17·3, 20·1) | 20·4 (18·6, 22·0) | 22·2 (20·0, 24·0) |
| 1 | 4 | 16·8 (15·9, 17·6) | 16·5 (15·8, 17·2) | 16·2 (15·5, 17·2) | 17·9 (16·8, 19·2) | 19·1 (17·2, 21·2) | 21·0 (19·6, 22·5) | 22·9 (21·0, 24·9) |
| 1 | 5 | 17·3 (16·6, 18·0) | 17·2 (16·5, 18·2) | 17·7 (16·6, 19·1) | 19·3 (18·0, 20·7) | 21·3 (19·7, 23·0) | 23·5 (21·4, 25·8) | 24·2 (21·8, 27·0) |
| 2 | 1 | 16·5 (15·9, 17·2) | 15·9 (15·4, 16·6) | 16·0 (15·3, 16·7) | 16·2 (15·5, 17·3) | 17·1 (16·5, 18·1) | 18·3 (17·2, 19·4) | 19·7 (18·3, 21·6) |
| 2 | 2 | 16·7 (16·3, 17·1) | 16·1 (15·7, 16·5) | 16·0 (15·5, 16·7) | 16·9 (16·3, 17·6) | 18·1 (17·2, 19·0) | 19·2 (18·5, 19·9) | 20·6 (19·5, 21·6) |
| 2 | 3 | 17·0 (16·2, 17·7) | 16·4 (15·8, 17·1) | 16·9 (16·3, 17·6) | 17·7 (16·9, 18·6) | 19·4 (18·3, 21·0) | 20·6 (19·5, 22·0) | 21·9 (20·2, 23·3) |
| 2 | 4 | 17·0 (16·4, 17·5) | 16·3 (15·7, 16·9) | 17·1 (16·1, 18·0) | 17·7 (16·7, 18·7) | 19·5 (18·1, 21·1) | 21·7 (20·0, 23·6) | 23·4 (21·2, 25·5) |
| 2 | 5 | 17·0 (16·3, 17·6) | 17·0 (16·1, 18·0) | 17·5 (16·5, 18·7) | 18·4 (17·4, 19·5) | 20·8 (19·4, 22·4) | 22·2 (20·6, 24·3) | 24·5 (22·2, 27·5) |
| 3 | 1 | 16·5 (16·0, 17·2) | 15·9 (15·4, 16·6) | 15·6 (15·0, 16·1) | 16·5 (15·8, 17·2) | 17·4 (16·2, 18·5) | 18·6 (17·4, 19·7) | 20·1 (18·6, 21·8) |
| 3 | 2 | 16·7 (16·1, 17·3) | 15·8 (15·4, 16·3) | 15·8 (15·4, 16·2) | 16·9 (16·3, 17·6) | 18·0 (17·3, 18·8) | 19·7 (18·7, 20·8) | 21·0 (19·8, 22·5) |
| 3 | 3 | 16·9 (16·4, 17·4) | 16·4 (16·0, 16·8) | 16·5 (15·8, 17·4) | 17·1 (16·4, 18·0) | 18·4 (17·6, 19·4) | 20·1 (18·8, 21·4) | 21·9 (20·4, 24·0) |
| 3 | 4 | 16·8 (16·1, 17·5) | 16·3 (15·4, 17·4) | 16·5 (15·9, 17·1) | 17·3 (16·6, 18·0) | 18·8 (17·6, 20·0) | 20·2 (18·7, 21·8) | 21·9 (20·7, 23·1) |
| 3 | 5 | 17·4 (16·8, 18·1) | 16·9 (16·3, 17·6) | 17·3 (16·2, 18·5) | 18·0 (16·8, 19·1) | 19·3 (17·4, 20·8) | 21·9 (20·4, 23·9) | 22·9 (20·7, 24·9) |
| 4 | 1 | 16·5 (15·9, 17·0) | 15·9 (15·5, 16·4) | 16·1 (15·3, 16·8) | 16·6 (15·8, 17·4) | 17·0 (16·1, 18·2) | 18·7 (17·7, 20·0) | 20·6 (19·2, 22·2) |
| 4 | 2 | 16·9 (16·4, 17·4) | 16·1 (15·6, 16·7) | 16·2 (15·7, 16·8) | 16·9 (16·1, 17·9) | 17·9 (16·9, 18·9) | 19·2 (18·1, 20·5) | 20·7 (19·2, 22·5) |
| 4 | 3 | 17·0 (16·4, 17·6) | 16·4 (15·9, 17·1) | 16·3 (15·8, 16·9) | 17·2 (16·3, 18·4) | 18·7 (17·3, 20·5) | 19·6 (18·7, 20·6) | 21·7 (20·0, 23·9) |
| 4 | 4 | 16·8 (16·3, 17·4) | 16·1 (15·6, 16·6) | 16·4 (15·9, 16·9) | 17·6 (16·5, 19·0) | 18·7 (17·6, 19·9) | 20·1 (18·7, 21·8) | 21·6 (20·2, 23·6) |
| 4 | 5 | 17·0 (16·5, 17·5) | 16·5 (15·9, 17·1) | 16·7 (16·1, 17·3) | 18·0 (17·1, 18·9) | 19·2 (18·3, 20·1) | 20·8 (19·4, 22·2) | 22·8 (21·3, 24·3) |
| 5 | 1 | 16·4 (16·0, 16·8) | 15·9 (15·5, 16·4) | 15·6 (15·0, 16·2) | 16·4 (15·9, 17·0) | 17·0 (16·4, 17·6) | 18·5 (17·5, 19·4) | 19·8 (18·8, 20·8) |
| 5 | 2 | 16·5 (15·9, 17·1) | 16·1 (15·5, 16·7) | 16·0 (15·2, 16·8) | 16·5 (15·8, 17·0) | 17·5 (16·8, 18·3) | 19·2 (18·4, 20·1) | 20·6 (19·6, 21·6) |
| 5 | 3 | 16·7 (15·9, 17·4) | 16·1 (15·3, 16·8) | 16·3 (15·6, 17·3) | 17·3 (16·2, 19·1) | 18·4 (17·2, 20·4) | 20·2 (18·1, 23·1) | 22·0 (19·7, 25·5) |
| 5 | 4 | 16·8 (16·2, 17·3) | 16·4 (15·8, 17·2) | 16·6 (15·9, 17·4) | 17·7 (17·0, 18·6) | 18·7 (17·7, 20·0) | 20·7 (19·3, 22·4) | 22·2 (20·5, 24·5) |
| 5 | 5 | 17·1 (16·7, 17·6) | 16·6 (16·2, 17·2) | 16·7 (16·1, 17·4) | 17·7 (16·8, 18·5) | 18·7 (17·5, 19·7) | 20·7 (19·5, 22·1) | 21·9 (20·8, 23·1) |
| Statistical significance of model interaction term SEIFA x PRS: p=0.17  Aim 1 focuses on describing the trends over time within PRS and SES strata and was not focused on estimating the outcomes at particular ages or waves. The estimates provided in these tables should not be used to make specific inferences to the population in general. | | | | | | | | |

**Supplementary Table 8: Estimated BMI (95% CI in brackets) used to generate Supplementary Figure 3; “BMI across childhood by family disadvantage (SEP) quintile (1=most, 5=least disadvantage), stratified by PRS quintile (1=lowest, 5=highest risk)”.**

|  |  | **Age** | | | | | | |
| --- | --- | --- | --- | --- | --- | --- | --- | --- |
| **SEP** | **PRS** | **2-3y** | **4-5y** | **6-7y** | **8-9y** | **10-11y** | **12-13y** | **14y+** |
| 1 | 1 | 16·6 (16·1, 17·1) | 16·1 (15·4, 16·9) | 16·1 (15·4, 17·1) | 17·2 (16·0, 19·0) | 17·8 (16·4, 19·7) | 19·5 (17·9, 21·4) | 20·2 (17·9, 23·3) |
| 1 | 2 | 16·7 (16·3, 17·1) | 16·1 (15·6, 16·7) | 16·0 (15·3, 16·9) | 16·8 (16·1, 17·6) | 17·7 (16·7, 18·6) | 19·3 (18·3, 20·3) | 21·2 (19·8, 22·4) |
| 1 | 3 | 17·3 (16·7, 17·8) | 16·7 (16·2, 17·4) | 17·1 (16·1, 18·3) | 18·3 (16·9, 20·0) | 19·9 (18·3, 21·7) | 21·4 (19·4, 23·5) | 23·4 (20·8, 25·7) |
| 1 | 4 | 16·9 (16·0, 17·9) | 16·4 (15·6, 17·5) | 16·7 (15·8, 17·8) | 18·0 (17·1, 19·0) | 19·7 (18·3, 21·2) | 21·5 (19·9, 23·1) | 23·0 (21·6, 24·4) |
| 1 | 5 | 17·1 (16·5, 17·6) | 17·0 (16·3, 17·9) | 17·6 (16·6, 18·8) | 18·6 (17·2, 19·9) | 21·3 (19·6, 23·4) | 23·1 (21·1, 25·2) | 24·7 (22·2, 27·3) |
| 2 | 1 | 16·4 (15·7, 17·1) | 16·0 (15·4, 16·4) | 15·6 (14·9, 16·1) | 16·4 (15·7, 17·2) | 17·5 (16·8, 18·3) | 18·5 (17·7, 19·5) | 20·7 (19·1, 22·9) |
| 2 | 2 | 16·7 (16·1, 17·3) | 15·9 (15·4, 16·4) | 16·0 (15·5, 16·7) | 16·8 (15·9, 17·7) | 18·0 (17·0, 18·8) | 19·3 (18·2, 20·3) | 19·9 (18·4, 21·3) |
| 2 | 3 | 16·7 (15·9, 17·6) | 16·3 (15·5, 17·3) | 16·6 (15·9, 17·4) | 17·7 (17·0, 18·7) | 19·0 (17·7, 20·4) | 20·2 (19·1, 21·3) | 22·2 (20·0, 24·6) |
| 2 | 4 | 16·8 (16·1, 17·4) | 16·1 (15·4, 17·0) | 16·7 (16·0, 17·4) | 17·6 (16·7, 18·5) | 18·8 (17·7, 20·2) | 20·2 (18·8, 21·7) | 21·7 (20·6, 23·0) |
| 2 | 5 | 17·6 (16·7, 18·5) | 17·3 (16·5, 18·0) | 17·8 (16·9, 18·7) | 18·9 (17·8, 20·2) | 20·2 (18·9, 21·4) | 22·4 (20·7, 24·9) | 24·0 (21·9, 26·1) |
| 3 | 1 | 16·3 (15·6, 17·0) | 15·7 (15·1, 16·3) | 15·6 (15·0, 16·2) | 16·2 (15·6, 16·9) | 16·9 (16·2, 17·6) | 18·8 (18·0, 19·8) | 19·9 (18·8, 21·1) |
| 3 | 2 | 16·6 (16·0, 17·3) | 16·2 (15·7, 16·6) | 15·8 (15·3, 16·2) | 16·8 (16·3, 17·3) | 17·7 (16·5, 18·9) | 19·1 (17·9, 20·3) | 20·7 (19·0, 22·2) |
| 3 | 3 | 16·8 (16·2, 17·6) | 16·4 (15·8, 17·0) | 16·4 (15·8, 17·2) | 16·8 (15·8, 17·8) | 18·6 (17·2, 20·0) | 19·7 (18·4, 20·8) | 20·8 (19·3, 22·0) |
| 3 | 4 | 16·9 (16·4, 17·3) | 16·4 (15·9, 16·9) | 16·3 (15·7, 17·2) | 17·6 (16·7, 18·8) | 18·6 (17·6, 19·9) | 20·1 (18·6, 22·1) | 22·2 (20·1, 24·8) |
| 3 | 5 | 17·0 (16·2, 17·8) | 16·7 (15·9, 17·4) | 16·9 (16·1, 17·6) | 18·4 (17·5, 19·6) | 19·7 (18·5, 21·2) | 21·9 (20·3, 24·3) | 22·7 (21·3, 24·4) |
| 4 | 1 | 16·6 (16·0, 17·2) | 16·0 (15·5, 16·5) | 15·6 (15·1, 16·0) | 16·4 (15·7, 17·1) | 16·9 (16·0, 17·7) | 18·1 (16·9, 19·2) | 19·8 (18·5, 21·5) |
| 4 | 2 | 16·8 (16·3, 17·3) | 15·9 (15·5, 16·4) | 16·2 (15·6, 17·0) | 16·6 (15·6, 17·8) | 18·0 (16·7, 19·6) | 19·4 (17·9, 21·0) | 20·6 (18·7, 22·6) |
| 4 | 3 | 16·8 (16·2, 17·4) | 16·2 (15·7, 16·8) | 16·2 (15·6, 16·8) | 17·0 (16·3, 17·8) | 18·0 (17·2, 19·0) | 19·8 (18·3, 21·4) | 22·1 (20·7, 23·5) |
| 4 | 4 | 16·9 (16·4, 17·4) | 16·5 (16·0, 17·1) | 17·0 (16·2, 17·8) | 18·0 (16·8, 19·2) | 19·2 (18·0, 20·3) | 21·6 (20·3, 23·2) | 23·0 (20·5, 26·1) |
| 4 | 5 | 17·0 (16·5, 17·5) | 16·7 (16·3, 17·3) | 16·7 (16·1, 17·4) | 17·6 (16·8, 18·8) | 19·1 (17·8, 20·3) | 20·7 (19·5, 22·1) | 22·2 (20·6, 23·7) |
| 5 | 1 | 16·4 (16·0, 16·7) | 15·9 (15·5, 16·2) | 15·8 (15·4, 16·3) | 16·3 (15·7, 17·0) | 17·2 (16·3, 18·2) | 18·8 (17·8, 19·8) | 19·8 (18·7, 20·8) |
| 5 | 2 | 16·9 (16·4, 17·3) | 16·2 (15·8, 16·5) | 16·0 (15·5, 16·5) | 16·9 (16·3, 17·5) | 17·8 (17·1, 18·5) | 19·3 (18·2, 20·2) | 20·9 (19·9, 21·9) |
| 5 | 3 | 16·6 (15·8, 17·2) | 16·0 (15·4, 16·5) | 16·0 (15·5, 16·5) | 16·7 (16·0, 17·3) | 18·1 (17·3, 18·9) | 19·4 (18·4, 20·6) | 21·0 (19·8, 22·5) |
| 5 | 4 | 16·8 (16·3, 17·3) | 16·1 (15·5, 16·7) | 16·3 (15·7, 17·2) | 17·2 (16·5, 18·2) | 18·4 (17·5, 19·6) | 20·2 (19·0, 22·0) | 21·9 (20·6, 23·1) |
| 5 | 5 | 16·9 (16·5, 17·5) | 16·4 (15·5, 17·0) | 16·6 (15·7, 17·3) | 17·6 (16·6, 18·5) | 19·0 (17·7, 20·1) | 20·5 (18·9, 21·8) | 22·4 (21·1, 24·0) |
| Statistical significance of model interaction term SEP x PRS: p=0.48  Aim 1 focuses on describing the trends over time within PRS and SES strata and was not focused on estimating the outcomes at particular ages or waves. The estimates provided in these tables should not be used to make specific inferences to the population in general. | | | | | | | | |

**Supplementary Table 9: Estimated BMI (95% CI in brackets) used to generate Main Figure 2; “Estimated BMI across adulthood by neighbourhood disadvantage (SEIFA) quintile (1=most, 5=least disadvantage), stratified by PRS quintile (1=lowest, 5=highest risk)”.**

|  |  | **Age category** | | | | | |
| --- | --- | --- | --- | --- | --- | --- | --- |
| **SEIFA** | **PRS** | **<30y** | **30-35y** | **35-40y** | **40-45y** | **45-50y** | **50y+** |
| 1 | 1 | 24·4 (22·4, 27·3) | 25·3 (23·6, 27·2) | 25·5 (24·2, 26·8) | 26·1 (24·6, 27·8) | 25·7 (23·6, 28·4) | 25·8 (22·2, 28·5) |
| 1 | 2 | 27·4 (23·2, 32·9) | 26·5 (24·2, 30·5) | 25·8 (23·9, 28·0) | 26·1 (23·9, 28·6) | 26·4 (24·6, 28·7) | 26·7 (23·1, 30·2) |
| 1 | 3 | 26·9 (23·6, 30·8) | 28·3 (26·3, 31·5) | 28·0 (26·3, 29·8) | 29·1 (27·0, 31·8) | 27·8 (25·8, 30·3) | 28·0 (25·2, 31·3) |
| 1 | 4 | 27·1 (23·2, 32·3) | 29·4 (26·7, 32·3) | 28·7 (26·7, 31·3) | 29·5 (27·6, 31·5) | 29·1 (26·5, 32·4) | 32·6 (25·6, 39·9) |
| 1 | 5 | 28·0 (26·3, 30·5) | 29·3 (27·2, 31·6) | 30·2 (28·5, 32·0) | 30·4 (28·1, 32·1) | 31·0 (28·0, 33·9) | 30·7 (26·5, 37·1) |
| 2 | 1 | 24·3 (20·6, 34·3) | 24·7 (23·2, 26·6) | 25·1 (24·0, 26·2) | 25·7 (24·4, 26·9) | 25·7 (23·7, 27·4) | 28·5 (25·5, 31·9) |
| 2 | 2 | 26·2 (23·6, 29·2) | 26·6 (24·3, 28·5) | 26·4 (24·7, 28·6) | 26·5 (25·3, 27·9) | 26·7 (25·0, 28·6) | 28·4 (24·9, 33·2) |
| 2 | 3 | 27·4 (24·9, 31·4) | 26·6 (24·3, 29·5) | 26·6 (25·2, 28·0) | 26·8 (25·2, 28·5) | 26·7 (25·0, 28·9) | 27·8 (24·4, 33·2) |
| 2 | 4 | 27·5 (24·7, 30·3) | 29·9 (27·7, 32·5) | 29·2 (27·7, 30·7) | 29·0 (27·3, 31·2) | 29·5 (27·3, 31·9) | 30·1 (25·7, 36·1) |
| 2 | 5 | 27·7 (26·1, 30·1) | 27·9 (25·9, 30·0) | 29·2 (26·5, 33·3) | 30·3 (27·8, 33·3) | 30·3 (26·7, 33·5) | 30·9 (26·5, 35·8) |
| 3 | 1 | 25·3 (21·2, 29·0) | 25·0 (23·2, 27·0) | 24·9 (23·2, 26·6) | 25·8 (24·0, 27·6) | 25·6 (23·3, 28·4) | 25·9 (22·1, 30·4) |
| 3 | 2 | 24·7 (22·7, 32·0) | 26·1 (24·0, 28·7) | 26·2 (23·9, 28·3) | 26·2 (24·7, 28·4) | 26·1 (23·9, 28·8) | 25·3 (23·1, 27·6) |
| 3 | 3 | 24·7 (22·4, 27·2) | 25·9 (23·3, 30·0) | 26·9 (24·7, 29·5) | 26·8 (25·2, 28·4) | 26·7 (24·7, 28·8) | 27·4 (24·5, 30·4) |
| 3 | 4 | 28·8 (25·0, 33·0) | 28·3 (26·6, 30·1) | 28·3 (26·7, 30·1) | 29·3 (27·4, 31·0) | 28·6 (27·0, 31·2) | 27·9 (23·6, 31·8) |
| 3 | 5 | 27·3 (25·7, 29·7) | 28·4 (26·0, 30·9) | 28·7 (27·2, 30·2) | 28·4 (26·4, 30·2) | 29·6 (27·4, 31·6) | 29·5 (24·8, 34·4) |
| 4 | 1 | 25·0 (21·3, 30·2) | 24·3 (22·5, 26·6) | 24·1 (22·9, 25·6) | 24·7 (23·1, 26·3) | 25·1 (23·3, 27·3) | 25·7 (23·6, 28·2) |
| 4 | 2 | 26·1 (21·1, 31·0) | 26·0 (23·7, 28·7) | 26·3 (24·5, 28·3) | 26·0 (23·5, 28·5) | 25·7 (23·4, 28·3) | 25·5 (22·6, 29·6) |
| 4 | 3 | 25·8 (22·9, 29·2) | 26·3 (24·4, 28·7) | 26·8 (25·1, 28·6) | 26·6 (24·9, 28·5) | 27·0 (25·3, 28·6) | 27·2 (24·1, 29·5) |
| 4 | 4 | 27·6 (25·1, 31·5) | 27·4 (25·0, 30·3) | 27·6 (26·2, 29·3) | 28·3 (26·7, 30·0) | 27·6 (24·7, 30·1) | 27·6 (24·1, 29·9) |
| 4 | 5 | 26·2 (22·6, 32·7) | 28·0 (25·8, 30·5) | 27·3 (25·6, 28·7) | 27·0 (24·9, 28·9) | 28·0 (25·7, 30·2) | 27·9 (25·4, 31·2) |
| 5 | 1 | 28·5 (22·2, 33·9) | 24·9 (22·9, 27·7) | 24·5 (23·3, 26·0) | 24·7 (23·4, 25·9) | 24·1 (22·4, 25·7) | 24·6 (21·6, 27·8) |
| 5 | 2 | 24·8 (21·3, 27·9) | 25·8 (22·9, 28·9) | 25·3 (23·1, 27·6) | 25·8 (23·5, 28·1) | 25·8 (23·6, 27·7) | 25·9 (23·5, 27·9) |
| 5 | 3 | 25·3 (20·2, 30·6) | 26·3 (24·3, 28·3) | 26·3 (24·3, 28·8) | 26·6 (24·9, 28·5) | 26·9 (24·9, 29·4) | 25·7 (22·8, 28·8) |
| 5 | 4 | 27·8 (21·8, 35·3) | 26·5 (24·8, 28·7) | 26·4 (24·5, 28·3) | 26·9 (25·2, 28·6) | 26·8 (24·5, 28·6) | 27·1 (24·7, 30·2) |
| 5 | 5 | 28·8 (24·5, 35·6) | 27·1 (24·7, 29·5) | 27·5 (25·3, 29·9) | 27·8 (25·8, 30·0) | 27·5 (25·1, 30·2) | 28·5 (24·5, 33·2) |
| Statistical significance of model interaction term SEIFA x PRS: p=0.64  Aim 1 focuses on describing the trends over time within PRS and SES strata and was not focused on estimating the outcomes at particular ages or waves. The estimates provided in these tables should not be used to make specific inferences to the population in general. | | | | | | | |

**Supplementary Table 10: Estimated BMI (95% CI in brackets) used to generate Supplementary Figure 8; “Estimated BMI across adulthood by family disadvantage (SEP) quintile (1=most, 5=least disadvantage), stratified by PRS quintile (1=lowest, 5=highest risk)”.**

|  |  | **Age category** | | | | | |
| --- | --- | --- | --- | --- | --- | --- | --- |
| **SEP** | **PRS** | **<30y** | **30-35y** | **35-40y** | **40-45y** | **45-50y** | **50y+** |
| 1 | 1 | 25·5 (22·4, 31·8) | 26·0 (23·8, 29·6) | 26·2 (24·8, 27·7) | 26·7 (24·5, 29·1) | 25·5 (23·7, 27·8) | 27·2 (24·4, 31·1) |
| 1 | 2 | 27·3 (24·7, 29·9) | 26·8 (24·7, 29·7) | 27·0 (24·5, 30·0) | 27·5 (25·5, 29·9) | 26·4 (24·3, 28·2) | 26·5 (24·5, 28·7) |
| 1 | 3 | 27·5 (24·3, 31·6) | 28·2 (25·9, 31·3) | 28·5 (26·5, 30·7) | 28·5 (26·4, 31·5) | 28·7 (26·3, 31·6) | 26·9 (20·5, 32·6) |
| 1 | 4 | 28·5 (24·5, 33·2) | 30·0 (26·5, 34·2) | 30·4 (27·9, 33·7) | 30·9 (28·4, 33·9) | 30·4 (27·6, 33·9) | 32·6 (26·2, 40·3) |
| 1 | 5 | 27·7 (26·2, 29·8) | 29·7 (27·9, 31·9) | 30·7 (29·0, 33·0) | 31·3 (29·0, 33·8) | 32·2 (29·1, 35·1) | 30·9 (25·5, 36·4) |
| 2 | 1 | 24·8 (21·8, 27·8) | 25·8 (24·3, 27·5) | 25·6 (24·3, 26·9) | 26·4 (24·4, 28·3) | 25·8 (23·4, 27·8) | 24·9 (20·8, 28·4) |
| 2 | 2 | 26·5 (22·3, 32·8) | 27·2 (24·6, 30·4) | 26·2 (24·8, 27·9) | 26·6 (24·7, 28·9) | 26·7 (24·2, 29·3) | 25·5 (22·5, 29·5) |
| 2 | 3 | 25·9 (23·7, 28·8) | 27·3 (25·4, 29·7) | 27·4 (25·2, 29·9) | 27·9 (26·1, 29·8) | 26·7 (24·9, 28·4) | 26·3 (22·9, 30·8) |
| 2 | 4 | 27·5 (25·4, 30·8) | 29·3 (26·6, 31·8) | 29·4 (27·5, 32·2) | 29·8 (28·0, 31·9) | 28·9 (26·9, 31·2) | 30·1 (27·8, 33·9) |
| 2 | 5 | 28·0 (26·2, 31·4) | 28·7 (26·8, 30·8) | 29·2 (27·7, 30·6) | 29·6 (27·2, 31·8) | 29·9 (27·8, 31·8) | 29·6 (25·7, 33·7) |
| 3 | 1 | 24·9 (22·4, 28·2) | 24·3 (23·1, 25·4) | 24·6 (23·4, 25·6) | 25·0 (23·4, 26·7) | 25·4 (23·2, 27·7) | 26·1 (23·7, 28·9) |
| 3 | 2 | 23·5 (21·1, 26·6) | 26·3 (24·2, 28·6) | 26·4 (24·4, 28·4) | 26·4 (24·4, 28·4) | 26·1 (23·6, 28·8) | 26·4 (23·9, 29·3) |
| 3 | 3 | 26·2 (22·0, 30·5) | 26·5 (24·0, 30·5) | 27·1 (25·0, 29·4) | 27·1 (25·3, 29·4) | 27·1 (24·8, 29·6) | 27·8 (25·4, 30·3) |
| 3 | 4 | 27·8 (24·5, 32·3) | 28·8 (26·4, 32·0) | 27·6 (26·3, 29·0) | 28·1 (26·7, 29·6) | 27·8 (25·3, 29·9) | 30·2 (25·6, 33·8) |
| 3 | 5 | 28·1 (23·8, 32·4) | 29·1 (26·8, 32·1) | 28·9 (26·7, 31·7) | 28·8 (27·0, 30·8) | 28·3 (25·5, 30·7) | 29·0 (26·1, 31·8) |
| 4 | 1 | 23·5 (20·1, 29·0) | 23·9 (22·2, 26·3) | 24·6 (23·1, 26·4) | 25·2 (23·6, 26·8) | 24·7 (23·1, 26·2) | 25·1 (22·1, 28·3) |
| 4 | 2 | 24·4 (21·8, 27·9) | 25·7 (23·2, 28·8) | 25·6 (23·3, 28·2) | 26·1 (23·9, 28·5) | 25·9 (23·6, 28·0) | 26·8 (23·2, 29·9) |
| 4 | 3 | 24·6 (22·3, 26·5) | 25·9 (23·9, 27·6) | 26·7 (25·1, 28·0) | 27·1 (25·6, 28·7) | 26·6 (24·8, 28·5) | 26·8 (24·5, 29·1) |
| 4 | 4 | 26·7 (21·6, 35·5) | 26·8 (24·9, 30·1) | 27·2 (25·0, 29·7) | 28·0 (26·7, 29·2) | 28·1 (25·9, 29·8) | 28·0 (25·3, 30·3) |
| 4 | 5 | 26·8 (25·2, 28·8) | 27·4 (25·4, 29·9) | 27·5 (25·2, 30·5) | 27·1 (25·1, 29·4) | 27·4 (25·7, 29·7) | 25·6 (21·5, 33·1) |
| 5 | 1 | 22·3 (18·1, 26·7) | 24·2 (21·8, 26·7) | 23·6 (22·3, 24·8) | 24·2 (22·9, 25·1) | 24·6 (22·9, 26·5) | 26·1 (23·2, 30·2) |
| 5 | 2 | 26·4 (21·4, 32·9) | 25·1 (21·9, 28·8) | 25·0 (22·7, 27·6) | 24·9 (22·8, 27·1) | 25·2 (23·1, 27·4) | 25·2 (23·4, 27·5) |
| 5 | 3 | NA | 24·4 (22·8, 26·1) | 25·1 (23·7, 26·6) | 25·5 (24·3, 26·9) | 26·6 (25·3, 27·9) | 26·2 (23·3, 29·0) |
| 5 | 4 | 25·4 (22·4, 27·4) | 26·1 (24·0, 28·2) | 26·2 (24·4, 28·2) | 26·8 (24·9, 28·9) | 26·4 (24·9, 27·8) | 26·2 (23·4, 30·1) |
| 5 | 5 | 24·6 (20·8, 28·0) | 25·4 (23·6, 27·0) | 26·5 (24·4, 28·4) | 27·1 (25·0, 29·1) | 27·0 (24·7, 29·5) | 27·2 (24·5, 31·2) |
| Statistical significance of model interaction term SEP x PRS: p=0.94  Aim 1 focuses on describing the trends over time within PRS and SES strata and was not focused on estimating the outcomes at particular ages or waves. The estimates provided in these tables should not be used to make specific inferences to the population in general. | | | | | | | |

**Supplementary Table 11: Estimated probability of overweight/obesity (95% CI in brackets) across childhood by neighbourhood disadvantage (SEIFA) quintile (1=most, 5=least disadvantage), stratified by PRS quintile (1=lowest, 5=highest risk).**

|  |  | **Age** | | | | | | |
| --- | --- | --- | --- | --- | --- | --- | --- | --- |
| **SEIFA** | **PRS** | **2-3y** | **4-5y** | **6-7y** | **8-9y** | **10-11y** | **12-13y** | **14y+** |
| 1 | 1 | 0·16 (0·06, 0·27) | 0·22 (0·10, 0·35) | 0·07 (0·01, 0·17) | 0·10 (0·01, 0·22) | 0·14 (0·04, 0·28) | 0·15 (0·05, 0·29) | 0·11 (0·00, 0·28) |
| 1 | 2 | 0·34 (0·20, 0·52) | 0·32 (0·17, 0·45) | 0·16 (0·06, 0·29) | 0·16 (0·05, 0·34) | 0·17 (0·03, 0·36) | 0·17 (0·02, 0·37) | 0·17 (0·03, 0·33) |
| 1 | 3 | 0·27 (0·15, 0·41) | 0·31 (0·18, 0·46) | 0·18 (0·06, 0·32) | 0·23 (0·10, 0·39) | 0·25 (0·10, 0·42) | 0·28 (0·09, 0·50) | 0·29 (0·11, 0·47) |
| 1 | 4 | 0·32 (0·10, 0·63) | 0·36 (0·18, 0·55) | 0·20 (0·06, 0·37) | 0·29 (0·12, 0·48) | 0·32 (0·07, 0·61) | 0·34 (0·15, 0·54) | 0·38 (0·16, 0·66) |
| 1 | 5 | 0·45 (0·29, 0·62) | 0·54 (0·38, 0·68) | 0·42 (0·25, 0·60) | 0·44 (0·28, 0·61) | 0·50 (0·34, 0·67) | 0·54 (0·35, 0·73) | 0·49 (0·29, 0·68) |
| 2 | 1 | 0·24 (0·12, 0·41) | 0·24 (0·11, 0·42) | 0·12 (0·03, 0·24) | 0·06 (0·01, 0·16) | 0·05 (0·01, 0·12) | 0·04 (0·00, 0·12) | 0·07 (0·00, 0·20) |
| 2 | 2 | 0·28 (0·15, 0·42) | 0·29 (0·15, 0·48) | 0·12 (0·03, 0·27) | 0·12 (0·03, 0·24) | 0·12 (0·02, 0·23) | 0·09 (0·02, 0·17) | 0·10 (0·02, 0·20) |
| 2 | 3 | 0·36 (0·17, 0·60) | 0·32 (0·17, 0·51) | 0·26 (0·13, 0·42) | 0·24 (0·11, 0·41) | 0·26 (0·12, 0·46) | 0·33 (0·19, 0·51) | 0·32 (0·15, 0·51) |
| 2 | 4 | 0·31 (0·17, 0·45) | 0·25 (0·13, 0·39) | 0·28 (0·13, 0·46) | 0·29 (0·13, 0·48) | 0·31 (0·11, 0·55) | 0·35 (0·18, 0·54) | 0·40 (0·17, 0·60) |
| 2 | 5 | 0·42 (0·22, 0·60) | 0·48 (0·27, 0·68) | 0·40 (0·21, 0·62) | 0·40 (0·25, 0·55) | 0·46 (0·29, 0·64) | 0·40 (0·22, 0·59) | 0·46 (0·29, 0·64) |
| 3 | 1 | 0·23 (0·09, 0·44) | 0·23 (0·11, 0·40) | 0·08 (0·01, 0·18) | 0·09 (0·02, 0·21) | 0·08 (0·01, 0·20) | 0·09 (0·01, 0·19) | 0·11 (0·01, 0·22) |
| 3 | 2 | 0·30 (0·15, 0·47) | 0·18 (0·07, 0·29) | 0·09 (0·02, 0·17) | 0·14 (0·03, 0·29) | 0·16 (0·06, 0·28) | 0·19 (0·08, 0·36) | 0·16 (0·05, 0·29) |
| 3 | 3 | 0·35 (0·21, 0·51) | 0·32 (0·18, 0·49) | 0·21 (0·08, 0·35) | 0·22 (0·10, 0·39) | 0·21 (0·10, 0·37) | 0·24 (0·10, 0·43) | 0·26 (0·07, 0·53) |
| 3 | 4 | 0·29 (0·14, 0·44) | 0·27 (0·10, 0·48) | 0·23 (0·10, 0·41) | 0·21 (0·09, 0·34) | 0·25 (0·12, 0·40) | 0·26 (0·08, 0·44) | 0·30 (0·16, 0·44) |
| 3 | 5 | 0·52 (0·37, 0·68) | 0·47 (0·32, 0·63) | 0·38 (0·19, 0·57) | 0·30 (0·12, 0·48) | 0·28 (0·08, 0·49) | 0·42 (0·26, 0·60) | 0·38 (0·20, 0·62) |
| 4 | 1 | 0·26 (0·10, 0·41) | 0·25 (0·12, 0·37) | 0·14 (0·03, 0·30) | 0·15 (0·04, 0·28) | 0·12 (0·03, 0·26) | 0·16 (0·04, 0·30) | 0·19 (0·06, 0·37) |
| 4 | 2 | 0·33 (0·15, 0·52) | 0·22 (0·09, 0·35) | 0·12 (0·04, 0·25) | 0·14 (0·04, 0·28) | 0·14 (0·02, 0·27) | 0·15 (0·04, 0·29) | 0·13 (0·00, 0·30) |
| 4 | 3 | 0·38 (0·24, 0·54) | 0·31 (0·19, 0·46) | 0·17 (0·05, 0·30) | 0·17 (0·04, 0·32) | 0·23 (0·07, 0·44) | 0·20 (0·07, 0·35) | 0·24 (0·07, 0·49) |
| 4 | 4 | 0·36 (0·21, 0·54) | 0·27 (0·15, 0·42) | 0·20 (0·08, 0·33) | 0·27 (0·10, 0·46) | 0·28 (0·13, 0·45) | 0·23 (0·10, 0·38) | 0·25 (0·08, 0·47) |
| 4 | 5 | 0·39 (0·24, 0·56) | 0·36 (0·21, 0·55) | 0·26 (0·15, 0·39) | 0·30 (0·13, 0·48) | 0·31 (0·16, 0·46) | 0·29 (0·14, 0·44) | 0·32 (0·16, 0·48) |
| 5 | 1 | 0·13 (0·05, 0·25) | 0·21 (0·09, 0·34) | 0·07 (0·01, 0·18) | 0·07 (0·01, 0·14) | 0·07 (0·00, 0·18) | 0·07 (0·01, 0·14) | 0·07 (0·00, 0·20) |
| 5 | 2 | 0·27 (0·12, 0·45) | 0·29 (0·13, 0·51) | 0·13 (0·02, 0·29) | 0·05 (0·00, 0·10) | 0·10 (0·02, 0·20) | 0·11 (0·02, 0·21) | 0·09 (0·02, 0·21) |
| 5 | 3 | 0·24 (0·09, 0·38) | 0·27 (0·12, 0·45) | 0·19 (0·08, 0·34) | 0·18 (0·06, 0·38) | 0·18 (0·06, 0·33) | 0·23 (0·08, 0·44) | 0·25 (0·09, 0·46) |
| 5 | 4 | 0·27 (0·10, 0·46) | 0·31 (0·13, 0·48) | 0·24 (0·11, 0·38) | 0·25 (0·08, 0·46) | 0·26 (0·13, 0·43) | 0·29 (0·13, 0·48) | 0·31 (0·15, 0·51) |
| 5 | 5 | 0·39 (0·22, 0·60) | 0·38 (0·19, 0·60) | 0·31 (0·15, 0·48) | 0·24 (0·10, 0·39) | 0·25 (0·10, 0·41) | 0·29 (0·07, 0·53) | 0·27 (0·06, 0·49) |
| Statistical significance of model interaction term SEIFA x PRS: p=0.69  Aim 1 focuses on describing the trends over time within PRS and SES strata and was not focused on estimating the outcomes at particular ages or waves. The estimates provided in these tables should not be used to make specific inferences to the population in general. | | | | | | | | |

**Supplementary Table 12: Estimated probability of overweight/obesity (95% CI in brackets) across childhood by family disadvantage (SEP) quintile (1=most, 5=least disadvantage), stratified by PRS quintile (1=lowest, 5=highest risk).**

|  |  | **Age** | | | | | | |
| --- | --- | --- | --- | --- | --- | --- | --- | --- |
| **SEP** | **PRS** | **2-3y** | **4-5y** | **6-7y** | **8-9y** | **10-11y** | **12-13y** | **14y+** |
| 1 | 1 | 0·26 (0·10, 0·41) | 0·29 (0·13, 0·47) | 0·15 (0·05, 0·32) | 0·19 (0·04, 0·44) | 0·16 (0·03, 0·37) | 0·21 (0·06, 0·42) | 0·12 (0·00, 0·32) |
| 1 | 2 | 0·27 (0·15, 0·42) | 0·27 (0·15, 0·42) | 0·12 (0·03, 0·24) | 0·12 (0·02, 0·25) | 0·13 (0·02, 0·27) | 0·16 (0·03, 0·32) | 0·20 (0·08, 0·32) |
| 1 | 3 | 0·42 (0·27, 0·58) | 0·41 (0·26, 0·56) | 0·28 (0·09, 0·52) | 0·33 (0·13, 0·58) | 0·36 (0·15, 0·60) | 0·36 (0·15, 0·57) | 0·37 (0·16, 0·56) |
| 1 | 4 | 0·33 (0·13, 0·58) | 0·34 (0·15, 0·56) | 0·22 (0·06, 0·41) | 0·31 (0·15, 0·48) | 0·39 (0·21, 0·60) | 0·38 (0·21, 0·56) | 0·40 (0·25, 0·58) |
| 1 | 5 | 0·45 (0·24, 0·67) | 0·48 (0·30, 0·66) | 0·39 (0·22, 0·58) | 0·37 (0·17, 0·55) | 0·49 (0·32, 0·66) | 0·53 (0·35, 0·72) | 0·50 (0·34, 0·69) |
| 2 | 1 | 0·19 (0·07, 0·34) | 0·23 (0·12, 0·37) | 0·06 (0·01, 0·14) | 0·05 (0·01, 0·12) | 0·10 (0·01, 0·20) | 0·04 (0·00, 0·13) | 0·17 (0·05, 0·38) |
| 2 | 2 | 0·32 (0·19, 0·49) | 0·23 (0·09, 0·36) | 0·13 (0·05, 0·25) | 0·13 (0·03, 0·24) | 0·15 (0·06, 0·25) | 0·13 (0·02, 0·26) | 0·06 (0·00, 0·15) |
| 2 | 3 | 0·33 (0·12, 0·56) | 0·30 (0·10, 0·58) | 0·21 (0·08, 0·37) | 0·26 (0·12, 0·46) | 0·22 (0·09, 0·37) | 0·26 (0·12, 0·42) | 0·32 (0·10, 0·55) |
| 2 | 4 | 0·31 (0·13, 0·56) | 0·27 (0·09, 0·48) | 0·29 (0·14, 0·46) | 0·29 (0·16, 0·45) | 0·27 (0·12, 0·45) | 0·25 (0·09, 0·41) | 0·30 (0·14, 0·48) |
| 2 | 5 | 0·54 (0·31, 0·74) | 0·52 (0·37, 0·67) | 0·46 (0·30, 0·63) | 0·46 (0·29, 0·65) | 0·43 (0·26, 0·60) | 0·48 (0·30, 0·67) | 0·49 (0·26, 0·73) |
| 3 | 1 | 0·18 (0·05, 0·36) | 0·14 (0·05, 0·30) | 0·08 (0·01, 0·22) | 0·07 (0·01, 0·18) | 0·05 (0·00, 0·12) | 0·10 (0·02, 0·21) | 0·08 (0·00, 0·22) |
| 3 | 2 | 0·29 (0·15, 0·48) | 0·23 (0·12, 0·37) | 0·09 (0·02, 0·22) | 0·11 (0·04, 0·24) | 0·11 (0·01, 0·22) | 0·13 (0·00, 0·27) | 0·12 (0·00, 0·26) |
| 3 | 3 | 0·33 (0·19, 0·49) | 0·26 (0·10, 0·44) | 0·20 (0·07, 0·39) | 0·19 (0·06, 0·35) | 0·24 (0·08, 0·48) | 0·24 (0·08, 0·43) | 0·19 (0·07, 0·33) |
| 3 | 4 | 0·32 (0·17, 0·47) | 0·27 (0·14, 0·40) | 0·18 (0·08, 0·33) | 0·24 (0·14, 0·38) | 0·24 (0·11, 0·40) | 0·23 (0·11, 0·38) | 0·32 (0·09, 0·62) |
| 3 | 5 | 0·40 (0·23, 0·60) | 0·39 (0·16, 0·64) | 0·33 (0·16, 0·52) | 0·33 (0·17, 0·50) | 0·32 (0·16, 0·47) | 0·38 (0·21, 0·58) | 0·33 (0·12, 0·58) |
| 4 | 1 | 0·21 (0·09, 0·33) | 0·24 (0·11, 0·38) | 0·07 (0·01, 0·16) | 0·11 (0·03, 0·23) | 0·09 (0·01, 0·25) | 0·05 (0·00, 0·14) | 0·09 (0·00, 0·25) |
| 4 | 2 | 0·34 (0·13, 0·63) | 0·25 (0·12, 0·39) | 0·17 (0·04, 0·39) | 0·10 (0·00, 0·28) | 0·13 (0·00, 0·37) | 0·15 (0·01, 0·36) | 0·13 (0·00, 0·31) |
| 4 | 3 | 0·32 (0·17, 0·49) | 0·33 (0·20, 0·51) | 0·21 (0·09, 0·35) | 0·17 (0·05, 0·32) | 0·16 (0·05, 0·31) | 0·24 (0·07, 0·43) | 0·30 (0·14, 0·49) |
| 4 | 4 | 0·34 (0·20, 0·50) | 0·34 (0·20, 0·50) | 0·31 (0·16, 0·47) | 0·29 (0·12, 0·45) | 0·32 (0·18, 0·48) | 0·38 (0·21, 0·58) | 0·40 (0·15, 0·66) |
| 4 | 5 | 0·40 (0·25, 0·60) | 0·43 (0·29, 0·61) | 0·25 (0·10, 0·40) | 0·22 (0·08, 0·38) | 0·22 (0·11, 0·36) | 0·22 (0·07, 0·42) | 0·29 (0·15, 0·43) |
| 5 | 1 | 0·16 (0·07, 0·27) | 0·25 (0·14, 0·36) | 0·10 (0·03, 0·19) | 0·06 (0·01, 0·13) | 0·09 (0·02, 0·18) | 0·12 (0·02, 0·28) | 0·10 (0·02, 0·23) |
| 5 | 2 | 0·31 (0·18, 0·44) | 0·32 (0·19, 0·45) | 0·11 (0·04, 0·20) | 0·09 (0·02, 0·19) | 0·10 (0·03, 0·20) | 0·10 (0·02, 0·22) | 0·13 (0·04, 0·25) |
| 5 | 3 | 0·21 (0·10, 0·35) | 0·23 (0·11, 0·37) | 0·12 (0·04, 0·22) | 0·08 (0·01, 0·17) | 0·16 (0·05, 0·28) | 0·14 (0·03, 0·26) | 0·14 (0·03, 0·29) |
| 5 | 4 | 0·25 (0·11, 0·43) | 0·23 (0·10, 0·38) | 0·18 (0·07, 0·31) | 0·17 (0·06, 0·30) | 0·21 (0·08, 0·34) | 0·22 (0·09, 0·36) | 0·23 (0·09, 0·39) |
| 5 | 5 | 0·33 (0·13, 0·52) | 0·39 (0·11, 0·67) | 0·29 (0·13, 0·46) | 0·27 (0·13, 0·45) | 0·34 (0·17, 0·52) | 0·29 (0·11, 0·51) | 0·30 (0·09, 0·51) |
| Statistical significance of model interaction term SEIFA x PRS: p=0.93  Aim 1 focuses on describing the trends over time within PRS and SES strata and was not focused on estimating the outcomes at particular ages or waves. The estimates provided in these tables should not be used to make specific inferences to the population in general. | | | | | | | | |

**Supplementary Table 13: Estimated probability of overweight/obesity (95% CI in brackets) across adulthood by neighbourhood disadvantage (SEIFA) quintile (1=most, 5=least disadvantage), stratified by PRS quintile (1=lowest, 5=highest risk).**

|  |  | **Age category** | | | | | |
| --- | --- | --- | --- | --- | --- | --- | --- |
| **SEIFA** | **PRS** | **<30y** | **30-35y** | **35-40y** | **40-45y** | **45-50y** | **50y+** |
| 1 | 1 | 0·36 (0·05, 0·66) | 0·46 (0·29, 0·69) | 0·47 (0·31, 0·68) | 0·47 (0·30, 0·68) | 0·52 (0·30, 0·80) | 0·62 (0·16, 0·92) |
| 1 | 2 | 0·48 (0·13, 0·89) | 0·48 (0·27, 0·74) | 0·45 (0·28, 0·65) | 0·52 (0·32, 0·77) | 0·61 (0·40, 0·83) | 0·71 (0·29, 0·99) |
| 1 | 3 | 0·50 (0·11, 0·89) | 0·64 (0·44, 0·89) | 0·65 (0·50, 0·85) | 0·75 (0·57, 0·93) | 0·70 (0·43, 0·93) | 0·85 (0·50, 1·00) |
| 1 | 4 | 0·57 (0·09, 0·95) | 0·75 (0·56, 0·98) | 0·70 (0·54, 0·88) | 0·76 (0·61, 0·91) | 0·77 (0·53, 0·97) | 0·91 (0·56, 1·00) |
| 1 | 5 | 0·85 (0·58, 1·00) | 0·76 (0·61, 0·92) | 0·83 (0·64, 0·98) | 0·83 (0·61, 0·98) | 0·79 (0·59, 0·96) | 0·82 (0·52, 1·00) |
| 2 | 1 | 0·23 (0·00, 0·64) | 0·40 (0·23, 0·59) | 0·46 (0·27, 0·68) | 0·50 (0·34, 0·69) | 0·56 (0·31, 0·81) | 0·70 (0·40, 1·00) |
| 2 | 2 | 0·54 (0·18, 0·97) | 0·65 (0·33, 0·98) | 0·60 (0·40, 0·83) | 0·59 (0·45, 0·75) | 0·55 (0·35, 0·72) | 0·63 (0·28, 0·98) |
| 2 | 3 | 0·70 (0·30, 0·99) | 0·53 (0·31, 0·77) | 0·57 (0·39, 0·78) | 0·55 (0·39, 0·73) | 0·57 (0·34, 0·77) | 0·64 (0·27, 0·96) |
| 2 | 4 | 0·68 (0·41, 0·97) | 0·78 (0·55, 1·00) | 0·74 (0·57, 0·90) | 0·71 (0·58, 0·88) | 0·68 (0·48, 0·88) | 0·69 (0·41, 0·96) |
| 2 | 5 | 0·82 (0·51, 1·00) | 0·70 (0·44, 0·96) | 0·72 (0·51, 0·93) | 0·78 (0·56, 0·97) | 0·78 (0·50, 0·98) | 0·87 (0·49, 1·00) |
| 3 | 1 | 0·54 (0·07, 0·99) | 0·45 (0·21, 0·71) | 0·44 (0·21, 0·71) | 0·54 (0·29, 0·80) | 0·50 (0·23, 0·79) | 0·54 (0·11, 0·87) |
| 3 | 2 | 0·37 (0·01, 0·99) | 0·57 (0·32, 0·88) | 0·61 (0·32, 0·93) | 0·53 (0·39, 0·76) | 0·50 (0·28, 0·77) | 0·46 (0·22, 0·74) |
| 3 | 3 | 0·46 (0·04, 0·98) | 0·55 (0·29, 0·89) | 0·63 (0·38, 0·88) | 0·65 (0·44, 0·87) | 0·61 (0·39, 0·83) | 0·61 (0·30, 0·90) |
| 3 | 4 | 0·79 (0·40, 0·99) | 0·76 (0·58, 0·94) | 0·73 (0·53, 0·92) | 0·78 (0·52, 0·97) | 0·74 (0·47, 0·97) | 0·70 (0·32, 0·99) |
| 3 | 5 | 0·76 (0·48, 0·98) | 0·72 (0·41, 0·96) | 0·75 (0·52, 0·96) | 0·73 (0·50, 0·93) | 0·79 (0·56, 0·98) | 0·68 (0·26, 0·99) |
| 4 | 1 | 0·45 (0·01, 0·94) | 0·35 (0·14, 0·69) | 0·35 (0·19, 0·56) | 0·39 (0·21, 0·63) | 0·39 (0·19, 0·64) | 0·49 (0·14, 0·92) |
| 4 | 2 | 0·64 (0·01, 0·99) | 0·57 (0·25, 0·97) | 0·61 (0·34, 0·91) | 0·55 (0·24, 0·89) | 0·51 (0·24, 0·78) | 0·47 (0·17, 0·82) |
| 4 | 3 | 0·56 (0·15, 1·00) | 0·55 (0·28, 0·89) | 0·59 (0·42, 0·79) | 0·62 (0·43, 0·84) | 0·67 (0·50, 0·88) | 0·79 (0·41, 1·00) |
| 4 | 4 | 0·76 (0·27, 1·00) | 0·66 (0·45, 0·91) | 0·74 (0·51, 0·95) | 0·73 (0·50, 0·94) | 0·62 (0·30, 0·91) | 0·78 (0·45, 0·97) |
| 4 | 5 | 0·68 (0·17, 1·00) | 0·63 (0·43, 0·88) | 0·67 (0·43, 0·91) | 0·67 (0·41, 0·91) | 0·70 (0·46, 0·93) | 0·70 (0·43, 0·91) |
| 5 | 1 | 0·73 (0·02, 0·99) | 0·44 (0·21, 0·77) | 0·39 (0·24, 0·58) | 0·34 (0·20, 0·52) | 0·35 (0·16, 0·59) | 0·40 (0·07, 0·78) |
| 5 | 2 | 0·59 (0·04, 0·99) | 0·56 (0·14, 0·95) | 0·52 (0·19, 0·90) | 0·53 (0·21, 0·88) | 0·55 (0·23, 0·86) | 0·57 (0·23, 0·86) |
| 5 | 3 | 0·38 (0·00, 0·87) | 0·60 (0·39, 0·82) | 0·57 (0·34, 0·82) | 0·60 (0·41, 0·80) | 0·66 (0·42, 0·92) | 0·58 (0·19, 1·00) |
| 5 | 4 | 0·63 (0·01, 0·99) | 0·55 (0·32, 0·84) | 0·58 (0·31, 0·90) | 0·61 (0·35, 0·88) | 0·63 (0·33, 0·90) | 0·72 (0·39, 0·98) |
| 5 | 5 | 0·77 (0·36, 0·99) | 0·68 (0·40, 0·97) | 0·65 (0·40, 0·93) | 0·65 (0·47, 0·86) | 0·56 (0·35, 0·84) | 0·48 (0·19, 0·81) |
| Statistical significance of model interaction term SEIFA x PRS: p=0.93  Aim 1 focuses on describing the trends over time within PRS and SES strata and was not focused on estimating the outcomes at particular ages or waves. The estimates provided in these tables should not be used to make specific inferences to the population in general. | | | | | | | |

**Supplementary Table 14: Estimated probability of overweight/obesity (95% CI) across adulthood by family disadvantage (SEP) quintile (1=most, 5=least disadvantage), stratified by PRS quintile (1=lowest, 5=highest risk).**

|  |  | **Age category** | | | | | |
| --- | --- | --- | --- | --- | --- | --- | --- |
| **SEP** | **PRS** | **<30y** | **30-35y** | **35-40y** | **40-45y** | **45-50y** | **50y+** |
| 1 | 1 | 0·43 (0·14, 0·92) | 0·49 (0·30, 0·79) | 0·55 (0·39, 0·80) | 0·56 (0·32, 0·72) | 0·44 (0·21, 0·66) | 0·62 (0·19, 0·94) |
| 1 | 2 | 0·69 (0·35, 0·98) | 0·66 (0·39, 0·97) | 0·63 (0·35, 0·98) | 0·69 (0·43, 0·95) | 0·54 (0·29, 0·75) | 0·57 (0·30, 0·81) |
| 1 | 3 | 0·63 (0·29, 0·98) | 0·63 (0·42, 0·89) | 0·65 (0·49, 0·83) | 0·71 (0·50, 0·95) | 0·71 (0·49, 0·99) | 0·53 (0·11, 1·00) |
| 1 | 4 | 0·69 (0·38, 0·98) | 0·80 (0·59, 0·93) | 0·81 (0·67, 0·94) | 0·85 (0·68, 0·98) | 0·78 (0·54, 0·97) | 0·80 (0·40, 1·00) |
| 1 | 5 | 0·85 (0·57, 1·00) | 0·82 (0·61, 0·99) | 0·87 (0·69, 0·99) | 0·88 (0·68, 1·00) | 0·83 (0·61, 1·00) | 0·70 (0·20, 1·00) |
| 2 | 1 | 0·42 (0·14, 0·69) | 0·53 (0·33, 0·78) | 0·49 (0·32, 0·71) | 0·55 (0·30, 0·81) | 0·51 (0·26, 0·76) | 0·44 (0·02, 0·84) |
| 2 | 2 | 0·51 (0·10, 0·98) | 0·71 (0·42, 0·99) | 0·59 (0·40, 0·82) | 0·63 (0·41, 0·87) | 0·61 (0·37, 0·86) | 0·50 (0·24, 0·79) |
| 2 | 3 | 0·47 (0·19, 0·94) | 0·69 (0·40, 0·94) | 0·66 (0·41, 0·92) | 0·69 (0·48, 0·89) | 0·62 (0·37, 0·87) | 0·60 (0·21, 1·00) |
| 2 | 4 | 0·71 (0·29, 0·99) | 0·77 (0·52, 0·99) | 0·73 (0·59, 0·88) | 0·74 (0·57, 0·91) | 0·73 (0·47, 0·94) | 0·93 (0·75, 1·00) |
| 2 | 5 | 0·80 (0·53, 1·00) | 0·74 (0·49, 0·96) | 0·78 (0·57, 0·96) | 0·77 (0·56, 0·97) | 0·82 (0·61, 0·99) | 0·73 (0·39, 0·97) |
| 3 | 1 | 0·44 (0·11, 0·81) | 0·40 (0·21, 0·65) | 0·42 (0·24, 0·63) | 0·43 (0·23, 0·67) | 0·52 (0·21, 0·85) | 0·56 (0·20, 0·89) |
| 3 | 2 | 0·20 (0·00, 0·58) | 0·56 (0·32, 0·83) | 0·60 (0·34, 0·89) | 0·54 (0·30, 0·84) | 0·60 (0·27, 0·92) | 0·65 (0·27, 0·94) |
| 3 | 3 | 0·65 (0·16, 0·99) | 0·53 (0·27, 0·80) | 0·63 (0·41, 0·88) | 0·62 (0·40, 0·85) | 0·63 (0·40, 0·86) | 0·82 (0·52, 1·00) |
| 3 | 4 | 0·74 (0·38, 0·99) | 0·79 (0·56, 0·99) | 0·71 (0·50, 0·94) | 0·72 (0·52, 0·92) | 0·69 (0·34, 0·98) | 0·81 (0·35, 1·00) |
| 3 | 5 | 0·73 (0·28, 1·00) | 0·77 (0·55, 0·97) | 0·76 (0·51, 0·97) | 0·71 (0·52, 0·89) | 0·68 (0·40, 0·93) | 0·72 (0·42, 0·94) |
| 4 | 1 | 0·40 (0·01, 1·00) | 0·34 (0·13, 0·61) | 0·39 (0·18, 0·65) | 0·48 (0·26, 0·73) | 0·43 (0·25, 0·66) | 0·45 (0·10, 0·78) |
| 4 | 2 | 0·46 (0·01, 0·99) | 0·52 (0·17, 0·93) | 0·51 (0·22, 0·84) | 0·56 (0·29, 0·84) | 0·49 (0·23, 0·75) | 0·63 (0·30, 0·88) |
| 4 | 3 | 0·40 (0·08, 0·78) | 0·52 (0·31, 0·75) | 0·60 (0·41, 0·80) | 0·66 (0·51, 0·84) | 0·62 (0·42, 0·85) | 0·67 (0·27, 0·97) |
| 4 | 4 | 0·50 (0·01, 1·00) | 0·56 (0·33, 0·84) | 0·68 (0·36, 0·97) | 0·75 (0·52, 0·96) | 0·70 (0·42, 0·95) | 0·81 (0·55, 0·99) |
| 4 | 5 | 0·76 (0·44, 1·00) | 0·64 (0·39, 0·92) | 0·62 (0·38, 0·89) | 0·70 (0·44, 0·93) | 0·65 (0·43, 0·92) | 0·50 (0·04, 0·94) |
| 5 | 1 | 0·24 (0·00, 0·97) | 0·33 (0·10, 0·64) | 0·30 (0·14, 0·53) | 0·29 (0·16, 0·44) | 0·35 (0·18, 0·61) | 0·50 (0·22, 0·83) |
| 5 | 2 | 0·41 (0·00, 0·98) | 0·38 (0·12, 0·75) | 0·48 (0·17, 0·82) | 0·42 (0·20, 0·67) | 0·46 (0·22, 0·68) | 0·42 (0·14, 0·80) |
| 5 | 3 | NA | 0·39 (0·13, 0·65) | 0·47 (0·30, 0·67) | 0·52 (0·36, 0·71) | 0·66 (0·49, 0·85) | 0·66 (0·32, 0·94) |
| 5 | 4 | 0·40 (0·04, 0·90) | 0·55 (0·30, 0·86) | 0·57 (0·36, 0·80) | 0·57 (0·34, 0·81) | 0·52 (0·33, 0·76) | 0·55 (0·20, 0·91) |
| 5 | 5 | 0·50 (0·00, 0·99) | 0·45 (0·23, 0·70) | 0·60 (0·35, 0·87) | 0·60 (0·37, 0·85) | 0·57 (0·30, 0·87) | 0·64 (0·19, 0·99) |
| Statistical significance of model interaction term SEIFA x PRS: p=0.93  Aim 1 focuses on describing the trends over time within PRS and SES strata and was not focused on estimating the outcomes at particular ages or waves. The estimates provided in these tables should not be used to make specific inferences to the population in general. | | | | | | | |
